# Apical periodontitis healing and postoperative pain following endodontic treatment with a reciprocating single-file, single-cone approach: A randomized controlled pragmatic clinical trial

**DOI:** 10.1371/journal.pone.0227347

**Published:** 2020-02-03

**Authors:** Fabricio Eneas Diniz de-Figueiredo, Laila Fernandes Lima, Giana Silveira Lima, Ludmila Smith Oliveira, Maria Amália Ribeiro, Manoel Brito-Junior, Marcos Brito Correa, Manoel Sousa-Neto, André Luis Faria e Silva

**Affiliations:** 1 Graduate Program in Health Sciences, Federal University of Sergipe, Aracaju, Sergipe, Brazil; 2 Graduate Program in Dentistry, Federal University of Sergipe, Aracaju, Sergipe, Brazil; 3 Graduate Program in Dentistry, Federal University of Pelotas, Pelotas, Rio Grande do Sul, Brazil; 4 Department of Dentistry, Federal University of Sergipe, Aracaju, SE, Brazil; 5 Department of Dentistry, State University of Montes Claros, Montes Claros, MG, Brazil; 6 Department of Restorative Dentistry and Dental Materials, Federal University of Pelotas, Pelotas, Rio Grande do Sul, Brazil; 7 Department of Restorative Dentistry, Dental School of Ribeirão Preto, University of São Paulo, Ribeirão Preto, SP, Brazil; National Taiwan University, School of Dentistry, TAIWAN

## Abstract

This trial assessed post-operative pain and healing of apical periodontitis following endodontic therapy with a reciprocating system compared to a crown-down technique with hand files and lateral compaction filling. One-hundred and twenty nonvital anterior teeth with apical periodontitis were randomly treated using either a reciprocating single file followed by matching-taper single-cone filling or a hand file and lateral compaction filling. Postoperative pain was assessed during the 7 days after the treatment, using a visual analogue scale and a verbal rating scale. Apical healing was assessed using the periapical index score after a 12-month follow-up. The hypothesis tested was that both protocols were equivalent and present similar effectiveness in healing periapical lesions. Data were analyzed through two one-sided tests, t-tests, as well as Mann-Whitney and Chi-squared tests (α = 0.05). Logistic regression was used to investigate the association of clinical and demographic factors with the success of treatment. Regardless of the assessment time, no difference in incidence (38%-43% at first 24h), intensity of postoperative pain, and incidence of flare-up (≈ 3%) was observed between the two endodontic protocols. Both protocols resulted in a similar healing rate of apical periodontitis. After 12 months, the success rate ranged from 73% to 78% and the difference between the treatments fell within the pre-established equivalence margin (-0.1; -0.41 to 0.2). Endodontic treatment combining a reciprocating single file with matching-taper single cone showed similar clinical effectiveness to the treatment using hand-file instrumentation and the lateral compaction filling.

## Introduction

The use of reciprocating motion with nickel-titanium (NiTi) files for root canal preparation was an important advance in endodontic therapy [1,2], primarily because it extends the lifespan of NiTi files [3] and reduces treatment time [4]. In addition, it enables filling the canal with a matching-taper single cone, which is simpler than other root canal filling techniques. Because of these and other advantages–reduction of technical sensitivity, which results in fewer procedural errors [5, 6]–the preparation of root canals with a reciprocating single-file system followed by the single-cone filling technique has become a popular protocol for endodontic therapy.

However, the shorter procedure time (mainly during instrumentation) obtained with a reciprocating file also can reduce the antimicrobial efficacy of solutions, which depends on the time [7] and volume [8] of irrigation to effectively disinfect the root canal. A reduced effect of irrigating solutions can compromise the reduction of the microbial content in the root canal system and, thus, hinder the apical periodontitis healing [9]. Furthermore, some studies have suggested that reciprocating NiTi files are associated with increased debris extrusion compared to rotary NiTi files [10], a drawback that can increase the likelihood of postoperative complications such as a higher incidence and severity of postoperative pain [11].

To the best of our knowledge, the long-term success of endodontic treatments performed with a protocol that includes reciprocating single-file root-canal preparation has not yet been investigated by either prospective or retrospective studies. Therefore, this randomized controlled pragmatic clinical trial aimed to evaluate the clinical effectiveness of endodontic treatment of anterior teeth with apical periodontitis performed with a reciprocating system (single file and single cone). The hypothesis tested was that the use of a reciprocating single-file, single-cone approach is at least as effective as the control for the outcome of apical periodontitis healing.

## Materials and methods

### Ethical approval

This study was approved by the Research Ethics Committee of studies involving human beings from the Federal University of Sergipe (protocol# 1.365.354).

### Protocol registration

This study was registered with the Brazilian Clinical Trials Registry under identification number RBR-7ZCP2N. The report follows the protocol established by the CONSORT statement [12].

### Trial design, settings and data collection

This study was performed from July 2016 to March 2019 in four public specialized dentistry services (endodontic treatment) in four different cities (i.e., Capela, Estância, Laranjeiras and Nossa Senhora do Socorro) located in the state of Sergipe (Brazil). This randomized pragmatic clinical trial utilized a two-arm, parallel design (1:1 allocation ratio equivalency). The endodontic treatments were performed either by combining a single-file and single-cone (SFSC) technique using the Reciproc system (experimental group) or by associating the crown-down hand files with the lateral compaction obturation (HFLC) technique (control group).

### Recruitment and eligibility criteria

The recruited participants were patients who were scheduled for an endodontic treatment in one of the public services where the study was performed. Patients with anterior teeth presenting pulp necrosis and radiographic evidence of apical periodontitis (symptomatic apical periodontitis, asymptomatic apical periodontitis, and chronic apical abscess) with a diameter greater than 2 mm were invited to participate in the study. Those who agreed to participate and signed the informed consent form were included in this study. Only one tooth per participant was included in the trial. Teeth with an immature apex, any radiographic evidence of root resorption, previously treated root canal or requiring extensive prosthetic rehabilitation were excluded. Participants presenting a pre-existing health or oral condition that placed them at risk during the trial, as well as those having generalized periodontal disease, and women who were pregnant or breastfeeding were also excluded.

### Sample size calculation

The sample size was calculated for the main outcome, defined as the mean periapical index score (PAI) difference between protocols of the apical lesion 1 year after treatment. Therefore, the sample size calculation was done for a continuous outcome, to be analyzed by a parametric test, and for equivalence trial. The calculation for similarity trial used an equivalence limit of 0.5, a standard deviation of 0.73 [13], type I error of 0.05, and a power test of 0.90. Furthermore, the sample was increased by 20% to compensate for any drop-out. The calculation was based the following formula: n = f(α, β/2) × 2 × σ^2^ / d^2^, where σ is the standard deviation, f(α, β) = [Φ^-1^(α) + Φ^-1^(β)]^2^, and Φ^-1^ is the cumulative distribution function of a standardized normal deviate. This resulted in 60 teeth per experimental condition. Although the calculation was performed for a parametric test, data of PAI did not show a normal distribution, and a non-parametric test was used (Wilcoxon test).

### Random sequence generation and allocation concealment

A random list for each study setting was created using the website www.sealedenvelope.com. The treatment to be performed on each patient was placed into opaque and sealed envelopes (30 envelopes per study setting) by a third party not involved in the study intervention. The dentists who performed the clinical procedures only opened the envelope at the moment of the intervention. Because this is an intervention study, clinicians could not be blinded for the procedures they were performing. The patients were not informed of which group they were allocated to.

### Study interventions

The endodontic treatments were performed by three endodontists with more than 5 years of clinical experience. One of these clinicians (F.E.D.F) performed the endodontic treatments in two study settings (Laranjeiras and Nossa Senhora do Socorro), while the treatments in the other services (Capela and Estância) were performed by L.F.M, and L.S.O., respectively. After the administration of local anesthesia and the placement of a rubber dam, the carious lesion was removed, and the access cavity was performed. All endodontic treatments were performed in a single session according to the randomization procedure.

#### HFLC technique

The initial glide path was established with stainless steel hand K-files (Dentsply Sirona Endodontics, Ballaigues, Switzerland)up to a size #15 and up to 2/3 of the estimated working length. Then, the crown-down technique was performed, initially with Gates-Glidden burs (Dentsply Maillefer, Ballaigues, Switzerland), which were used in a step-down manner to enlarge the orifice, prepare the cervical and middle-thirds of the canal, and provide straight-line access to its apical third. The apical foramen was located by using an electronic apex locator (RomiApex A-15 Romidan, Kiryat Ono, Israel), and the working length established 1.0 mm short of its “0.0” reading. The proper apical limit of root canal treatment is still a controversial matter, and we decided to determine the instrumentation length 1.0 mm short of the “0.0” reading of the apex locator because this approach ensures that instrumentation procedures was not beyond the apex. Apical preparation was then performed using ISO stainless steel hand files (Dentsply Sirona Endodontics, Ballaigues, Switzerland), starting with the selection of the first file to bind at the working length. The final instrumentation file was set at 3 sizes larger than the first file used. The lateral compaction obturation technique was used to fill the canals. A .02 taper gutta-percha cone was selected according to the master apical file and was prefitted into the canal at the working length. After the canal was dried with paper points (Dentsply Sirona Endodontics, Switzerland), the master cone was lightly coated with an epoxy resin-based sealer (AH Plus, Dentsply DeTrey, Konstanz, Germany) and placed into the canal down to the working length. Lateral compaction was performed with finger spreaders (Dentsply Sirona Endodontics, Switzerland) and accessory cones (Dentsply Sirona Endodontics, Switzerland) chosen according to the final root canal dimensions. The excess filling material was then removed with a heated instrument and the access cavity was sealed with glass-ionomer (Dentsply, Petrópolis, RJ, Brasil).

#### SFSC technique

Root canal preparation was performed with Reciproc instruments (VDW GmbH, Munich, Germany). The Reciproc file was selected based on a preoperative radiograph and root canal space. If the canal was partially or completely invisible on the radiograph, an R25 file was selected. Otherwise, a #30 or #20 hand file was inserted passively to 2/3 of the estimated working length. An R50 file was selected whenever a #30 hand file reached this length, and an R40 file was used whenever the 2/3 were reached by a #20 hand file. R25 was selected for narrow canals. The Reciproc instrument was introduced into the root canal with a slow in-and-out pecking motion, which did not exceed 3–4 mm in amplitude. After three in-and-out movements, the file was pulled out of the canals to clean the flutes. When the instrument reached 2/3 of the estimated working length, the foramen was located by using an electronic apex locator (RomiApex A-15, Romidan, Kiryat Ono, Israel), and the actual working length was established 1.0 mm short of the “0.0” electronic reading. Finally, the Reciproc instrument was then reused in the same manner until the working length was reached. After canal preparation, a matching-taper single gutta-percha cone (VDW GmbH, Munich, Germany) was selected according to the file used to instrument the canal. The canal was dried with sterile paper points (Dentsply Sirona Endodontics, Switzerland), and the selected cone was lightly coated with AH plus sealer and placed into the canal down to the working length. The excess filling material was then removed with a heated instrument, and the access cavities were sealed with glass-ionomer (Dentsply, Petrópolis, RJ, Brasil)

All teeth from both groups were submitted to the following procedures: irrigation with 2.5% sodium hypochlorite after each instrumentation cycle; canal patency by passing a stainless steel K-file ≤ #15 approximately 1.0 mm beyond the working length also after each instrumentation cycle; smear layer removal with 17% EDTA for 3 minutes; final irrigation with 2,5% sodium hypochlorite and final restoration with composite resin. Pre- and post-operative radiographs were taken with Kodak UltraSpeed #2, D sensitivity film (Kodak, São Paulo, SP, Brazil), processed manually by the time/temperature method. The long cone parallel technique was used, by employing X-ray holders (Endo Rh plus, Indusbello, Londrina, PR, Brazil), which were placed on the 30.5 x 40.5 mm size film, parallel to the long axis of the tooth and perpendicular to the X-Ray.

### Calibration of evaluators and evaluations

Thirty periapical radiographs of endodontically treated teeth not included in the study were digitized and used for calibration procedures by two independent and blinded evaluators. The evaluators (M.A.R. and M.B.J.) are endodontists with more than 15 years of clinical experience. The evaluation of such radiography was repeated until intra- and inter-evaluator agreement was obtained with a Kappa coefficient higher than 0.80.

Apical periodontitis was classified using the initial radiography, and according to the Periapical Index (PAI) with one of the following scores [14]: 1—Normal periapical structures; 2—Small changes in bone structure; 3—Changes in bone structure with some mineral loss; 4—Periodontitis with well-defined radiolucent area; and 5—Severe periodontitis with exacerbating features. Bitewing film holders were used to standardize the position of radiographs. Silicone impression material (Optosil Comfort, Heraeus Kulzer, Hanau, Germany) was placed on the film holder, and impressions of the tooth undergoing treatment were taken and used to place the device in the same position during the follow-up evaluation.

The postoperative pain reported by patients was recorded using both a visual analog scale (VAS) and a verbal rating scale (VRS). For the VAS, the patient set her/his pain level by pointing (with a pen) along a 10-cm continuous line between two endpoints (ranging from the absence of pain to unbearable pain). The distance between the marking and the border corresponding to the absence of pain was recorded. The peak of pain at the first 24 h, as well the pain reported at 24 h, 72 h and 7 days after the end of the endodontic treatment were recorded. Peak of pain was defined as the most intense pain felt by the patient. The participants received a form with VAS and VRS in after the endodontic intervention. They returned the forms in the second appointment that were scheduled 7 days after the intervention. Postoperative pain was also scored according to the VRS, where 0 = none, 1 = mild, 2 = moderate, 3 = considerable, 4 = severe, and 5 = unbearable. The latter scale was used to assess the incidence of postoperative pain (scores differing from 0). The occurrence of flare-up was recorded when the patients presented severe pain and swelling following the endodontic treatment [15].

The patients were recalled after 12-months. New periapical radiographs were taken, and the lesions were reclassified according to PAI. Inter-evaluator kappa coefficients at preoperative and 12-month analysis were 0.86 and 0.81, respectively. Discrepancies between the scores were solved by concordance between the evaluators. Then, the teeth were classified as healed (scores 1 or 2); healing (reduced score compared to baseline, but higher than 2); or not healed (teeth that presented the same or worst PAI score than that observed at baseline). Teeth that were clinically asymptomatic–defined by absence of pain, tenderness to percussion and/or palpation, sinus tract, or soft tissue swelling–and presented a PAI score of 1 or 2 were classified as “success”. The pragmatic aspects of the present trial were scored using the revised version of the pragmatic-explanatory continuum indicator summary (PRECIS-2) [16]. This tool consisted of nine domains scored on a 5-point Likert continuum (from 1 = very explanatory “ideal conditions” to 5 = very pragmatic “usual care conditions”) aiming to determine the extent to which a trial is explanatory or pragmatic (applicable in the “real-world”).

### Statistical analysis

Baseline demographic data of the participants and clinical characteristics of the teeth included in the study were categorized, and the absolute and relative frequencies were calculated (except for PAI scores, final preparation size scores, age and follow-up time, for which either mean and standard deviation or median and 1^st^ and 3^rd^ quartiles).

The Wilcoxon Rank-Sum test for equivalence using two one-sided tests (TOST) procedure [17] was used to assess if, 12 months after the treatment, the 90% confidence interval of the mean PAI score treatment difference fell within the pre-established equivalence limit (main outcome of interest) [18]. For all other outcomes, traditional two-sided superiority analyses were used. The association between treatment protocol and changes on periapical status was analyzed by Fisher`s exact test. The success rates for each treatment protocol were calculated, and differences were analyzed by Chi-squared testing. Univariate logistic regression was used to determine any association between explanatory variables and the treatment’s success rate. Factors with a p-value < 0.1 were included in a multivariate analysis. Odds ratios and 95% confidence intervals were calculated.

For all VAS data, normal distribution and possible differences between the treatments were assessed through the Shapiro-Wilk test and t-test, respectively. Data from VRS were analyzed through the Wilcoxon rank-sum test. The incidence of postoperative pain and flare-up was calculated, and Chi-squared tests were used to assess any possible association between treatment and incidence. Differences between the treatments regarding the incidence and intensity of postoperative pain were calculated, as well as the confidence intervals at 95%. The level of significance was set at 95% for all analyses, except for the equivalence tests (90%).

### Sensitivity analysis

Data were analyzed with both the per-protocol and Intention-to-treat (ITT) methods to evaluate if the loss to follow-up affected the results and conclusions of the study. In the per-protocol analyses, only the data of participants who returned for the 12-month follow-up visit was used. In the ITT analyses, data from all randomized patients were included in the analyses. The “last observation carried forward” method was used to replace data of participants that did not attend the 12-month evaluation visit.

## Results and discussion

**Fig 1:**
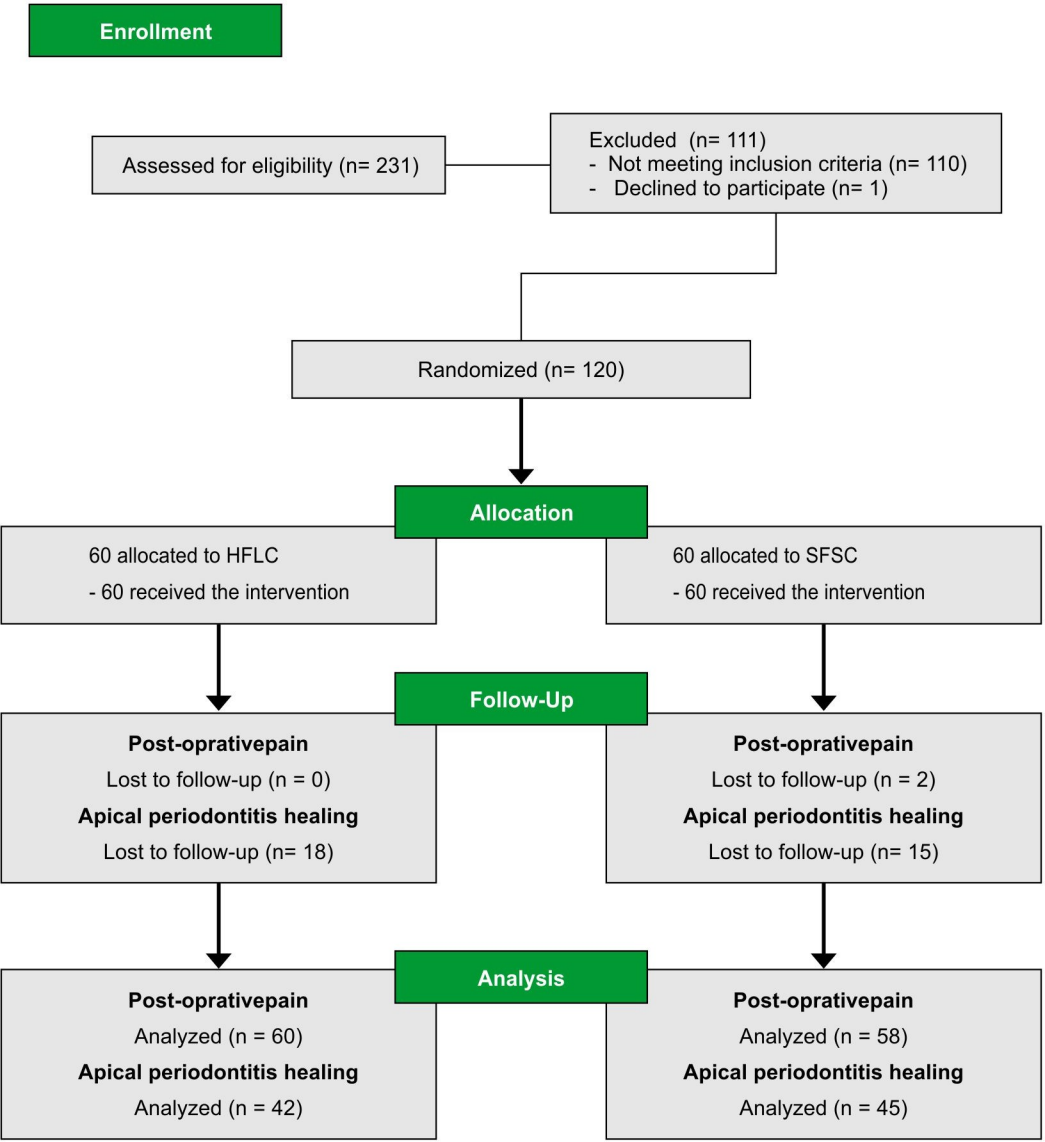
Flow chart diagram. HFLC–Hand-file and lateral compaction. SFSC–Single-file and single-cone.

The participants’ flow diagram in the different phases of the study is shown in Fig 1. Out of the 120 participants enrolled in the study, 33 did not attend the 12-month follow-up visit (15 in the SFSC group and 18 in the HFLC group) resulting in a drop-out rate of 27.5%. The analyses of missing data revealed that the drop-out was completely at random (Little’s MCAR test, p = 0.71) with no difference between the treatments (Chi-square, p = 0.68).

Baseline demographic and clinical characteristics of the 87 participants attending the 12-month follow-up appointment are presented in Table 1. The hand instrumentation protocol resulted in a larger (Mann-Whitney test, p < 0.0001) apical preparation size (0.6–0.55/0.80) than that performed with Reciproc files (0.5–0.5/0.5). Median– 1st and 3rd quartiles.

**Table 1:** Demographic and clinical baseline characteristics of participants included in the Periapical healing analyses 12-months after treatment.

Characteristics	Treatment	
	HFLC (n = 42)	SFSC (n = 45)
Age^a^(year-old)	36.9 (14.2)	34.2 (13.0)
Race^b^		
White	9 (21.4%)	6 (13.3%)
Black	7 (16.7%)	5 (11.1%)
Mixed	26 (61.9%)	34 (75.6%)
Gender^b^		
Male	16 (38.1%)	10 (22.2%)
Female	26 (61.9%)	35 (77.8%)
Tooth^b^		
Mandibular	7 (16.7%)	5 (11.1%)
Maxillary	35 (83.3%)	40 (88.9%)
Sinus tract^b^		
Present	14 (33.3%)	14 (31.1%)
Absent	28 (66.7%)	31 (68.9%)
PAI scores^c^		
	4.0 (3.0/4.0)	4.0 (3.0/4.0)
Study Setting		
Laranjeiras	22 (36.7%)	21 (35.0%)
N.S. do Socorro	21 (35.0%)	20 (33.3%)
Estância	13 (21.7%)	13 (21.7%)
Capela	4 (6.7%)	6 (10.0%)

PAI, Periapical Index; HFLC, Hand-file and lateral compaction technique; SFSC, Single-file and single-cone technique.

^a^ Means (Standard deviation).

^b^. n (%).

^c^. Medians (1^st^/ 3^rd^ quartiles).

Results for changes on PAI scores and periapical status are displayed in Table 2. The confidence interval for the mean difference in PAI score after the 12-month follow-up did not exceed the 0.5-bound pre-established as the equivalency limit, and both treatment protocols were effective in reducing PAI scores. No significant difference between the protocols in the distribution of periapical status changes was also observed.

**Table 2:** Results for changes on periapical condition during the follow-up time.

Treatment	F/U time (Mean, (SD), days)	PAI score (1–5) (Mean (SD))			Periapical status at 12-month F/U (n)			Success rate (95% CI)
		Baseline	12-month F/U	p-value	Healed	Healing	Not Healed	
HFLC (n = 42)	388.4 (33.6)	3.86 (0.75)	1.90 (0.84)	< 0.001^a^	31	9	2	0.73 (0.58/0.86)
SFSC (n = 45)	386.4 (25.2)	3.67 (0.76)	1.80 (0.89)	< 0.001^a^	35	8	2	0.78 (0.63/0.88)
Mean difference (90% CI)	-	-0.19 (-0.42/0.04)	-0.10 (-0.41/0.21)	-	-	-	-	-
p-value	0.75^b^	0.23^c^	0.02^c^	-	0.91^d^			0.66^d^

F/U, follow-up; SD, standard deviation; CI, Confidence Interval; HFLC, Hand-file and lateral compaction technique; SFSC, Single-file and single-cone technique, PAI–Periapical Index Score; Healed = PAI < 2; Healing = Teeth that presented improved PAI score but did not reach score < 2; Not Healed = teeth that presented same or worst PAI score than that observed at baseline. PAI–Periapical Index Score

^a^—Paired Wilcoxon signed-rank test

^b^—Independent T test

^c^—Wilcoxon Rank-Sum location difference test for equivalence using two one sided tests–TOST—(equivalency limit of -0.5 to 0.5 units on the PAI scale)

^d^—Chi-square test.

Multivariate analyses included participants’ age (as a continuous variable), gender, presence of sinus tract; extension, taper and overall quality of root canal obturation, sealer extrusion through the apical foramen, baseline PAI score and tooth location (maxillary or mandibular). The variables operator, study settings, race, final apical diameter, filling homogeneity, sealer extrusion, and participants’ income presented p-values > 0.1 in the univariate regression model and were excluded from the multivariate model.

Teeth presenting baseline PAI scores 4 and 5 were significantly associated with a lower chance of treatment success. No other factor significantly affected the success rate (Table 3).

**Table 3:** Results of the multivariate logistic regression model predicting the success rates of periapical periodontitis following the root canal treatment.

Independent variables	Crude OR (95% CI)	Adjusted OR (95% CI)	^a^P-value
Treatment (Ref.: HFLC)	0.85 (0.32–2.29)	0.78 (0.23–2.66)	0.69
Gender (Ref.: Male)	0.84 (0.29–2.42)	0.49 (0.12–2.0)	0.32
Baseline PAI score (Ref.: 3)	-	-	-
PAI = 4	0.11 (0.02–0.55)	0.06 (0.01–0.39)	0.003
PAI = 5	0.13 (0.02–0.80)	0.09 (0.01–0.74)	0.025
^b^Filling Extension (Ref.: Ideal)	0.76 (0.26–2.24)	0.2 (0.01–5.99)	0.35
^b^Filling Taper (Ref.: Ideal)	0.64 (0.13–3.25)	0.22 (0.01–8.19)	0.41
^b^Filling quality (Ref.: Deficient)	-		
Perfect	0.43 (0.05–3.85)	3 (0.04–236.4)	0.623
Satisfactory	0.38 (0.04–3.55)	0.42 (0.03–6.29)	0.527
Age (continuous variable)	0.99 (0.95–1.02)	0.96 (0.91–1.01)	0.11
Tooth position (Ref.: Maxilla)	1.02 (0.25–4.18)	0.21 (0.02–1.97)	0.14
Sinus tract (Ref.: Absent)	0.6 (0.19–1.84)	0.51 (0.14–1.94)	0.31

OR,Odds Ratio; CI, Confidence Interval; Ref., Reference; HFLC, Hand File, Lateral Compaction technique. PAI, Periapical Index Score.

^a^ Wald’s Test.

^b^ Criterions for root canal obturation quality parameters defined by a prior study [19] were adopted.

**Fig 2:**
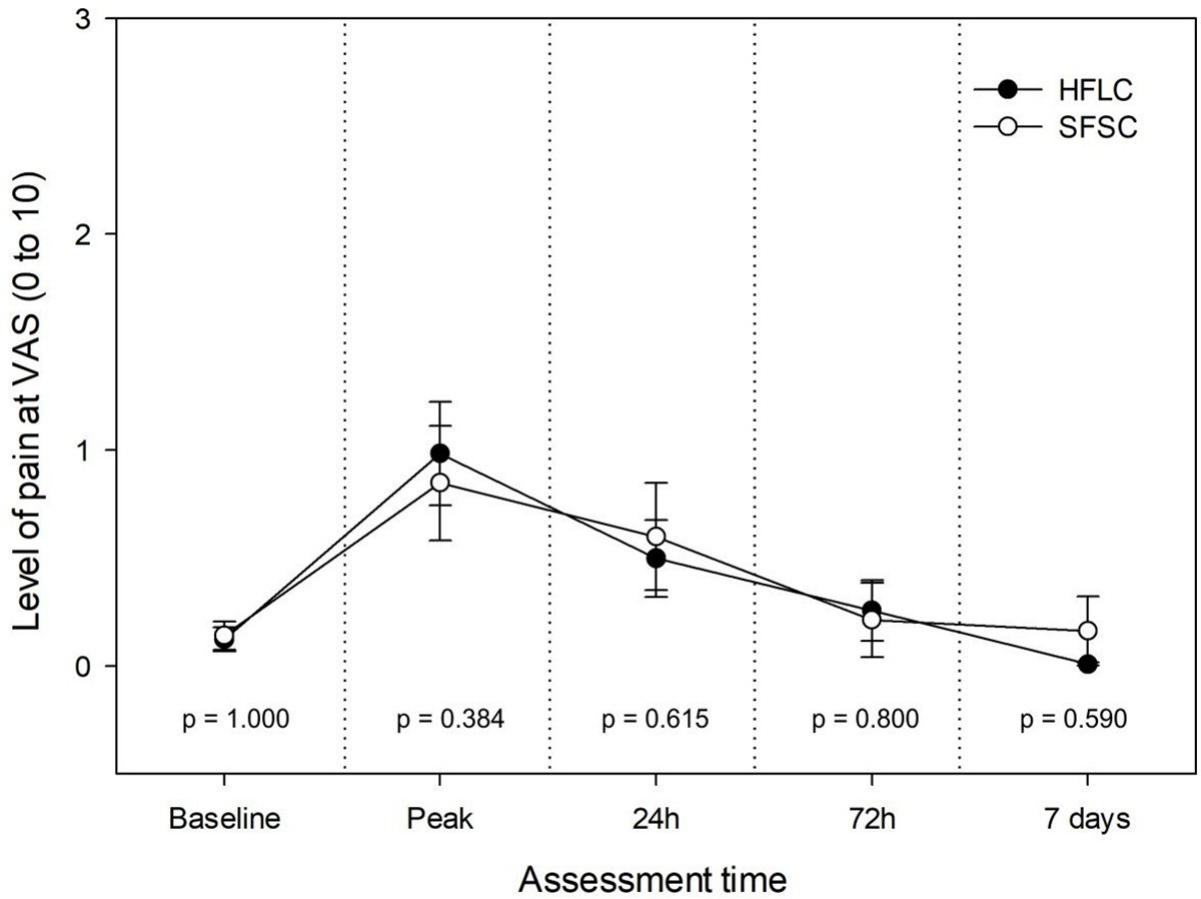
Baseline and post-operative pain intensity measured using visual analogue scale according to the assessment time and endodontic protocol. Circles indicate the means and bars represent the standard error. P-values calculated using the Mann-Whitney test. HFLC–Hand-file and lateral compaction; SFSC–Single-file and single-cone; VAS–Visual analogue scale. Peak = most intense pain felt by the patient in the first 24 hours after the treatment.

**Fig 3:**
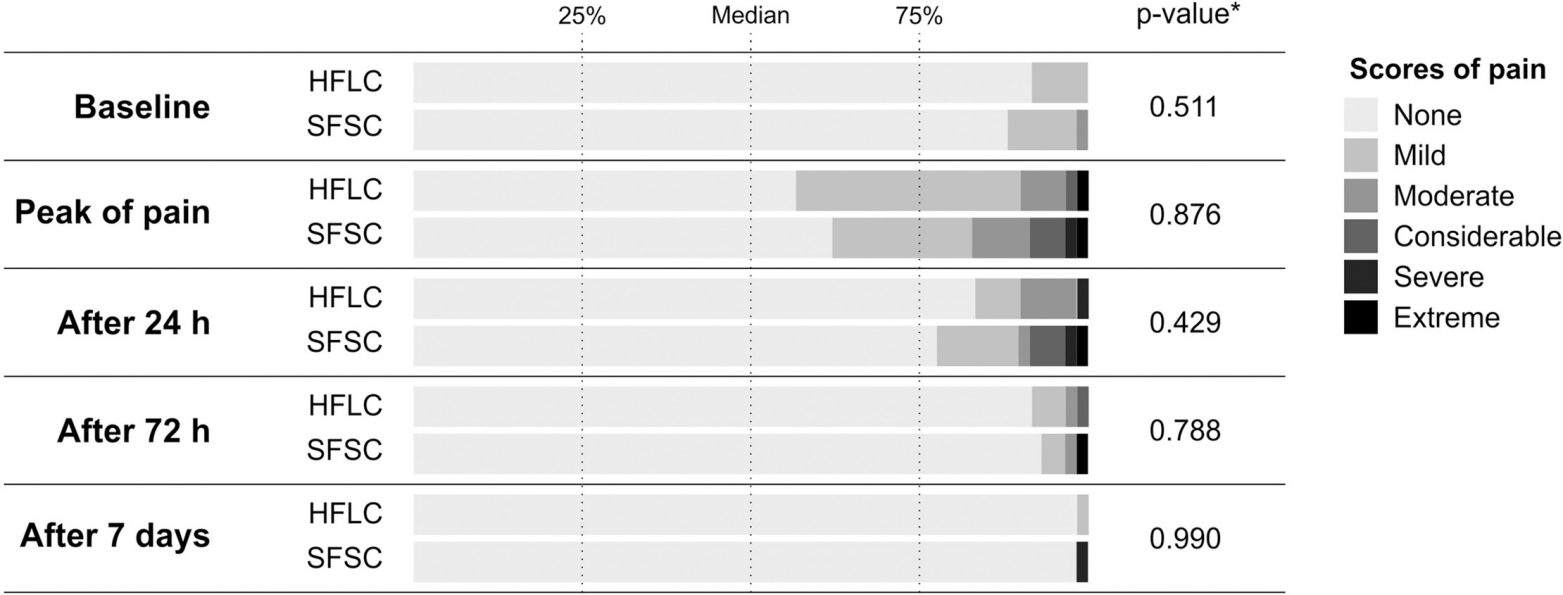
Distribution of scores reported by patients using a verbal rating scale regarding postoperative pain according to the time of assessment and treatment protocol. *Calculated using the Chi-square test. HFLC–Hand-file and lateral compaction; SFSC–Single-file and single-cone. Peak of pain = most intense pain felt by the patient in the first 24 hours after the treatment.

**Fig 4:**
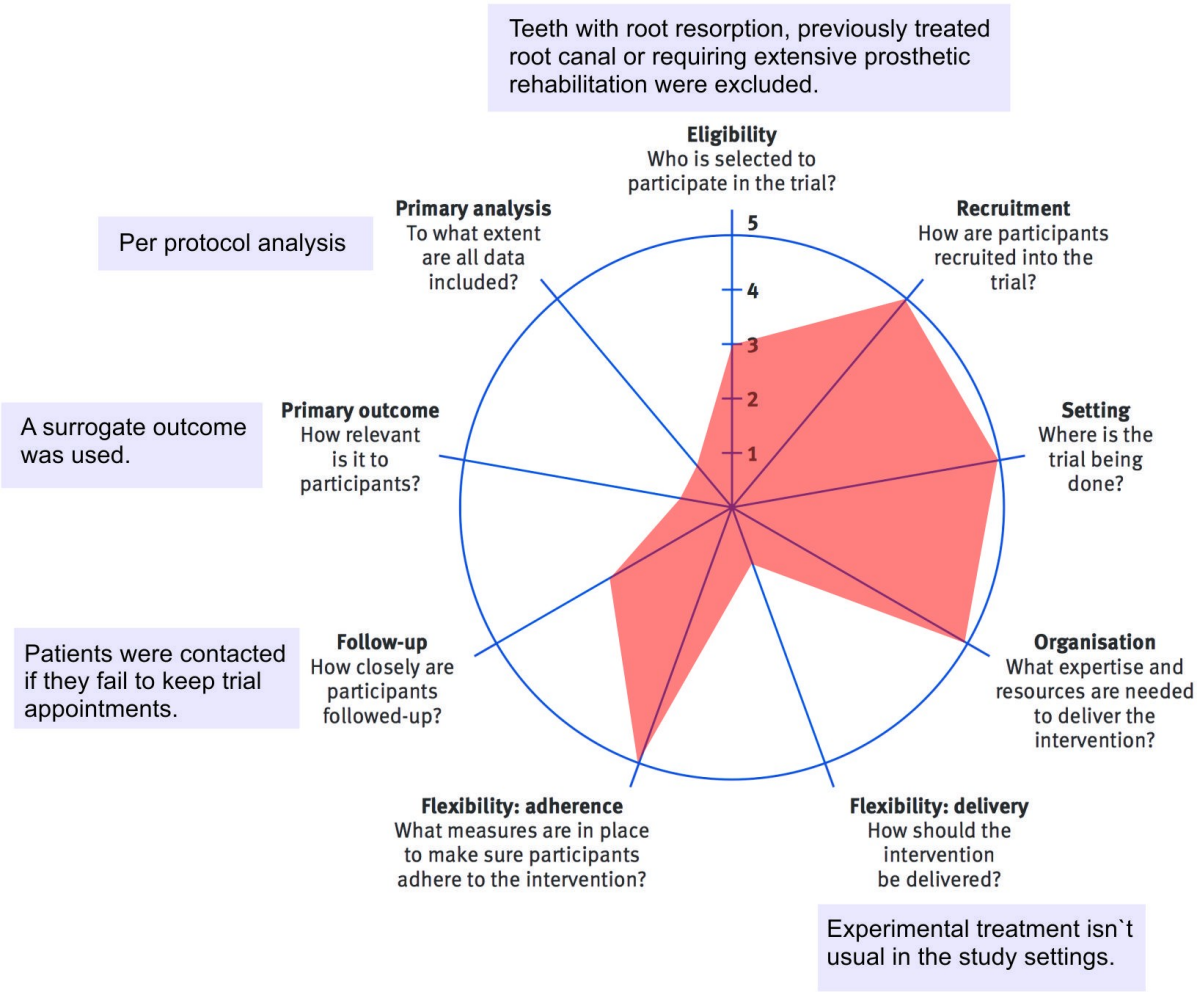
Pragmatism assessment of the trial using the pragmatic-explanatory continuum indicator summary (PRECIS2) diagram tool.

Two participants allocated to SFSC did not return the questionnaire used to assess post-operative pain and were excluded from the analyses. We observed no difference between the two endodontic protocols regarding clinical and demographic characteristics at baseline (p > 0.05), including the intensity (Figs 2 and 3) and incidence of pre-operative pain (Table 4). Irrespective of the assessment time, we observed no difference between the treatments regarding the incidence and intensity of pain. Furthermore, a low level of pain was reported at all assessment times and only 2 cases of flare-up were observed for each endodontic protocol. The study design was scored with 29-points (9 to 45 scale) using the PRECIS-2 tool (Fig 4).

**Table 4:** Results for incidence of postoperative pain using VRS and flare-up according to assessment time and protocol of endodontic treatment.

	HFLC (n = 60)	SFSC (n = 58)	Relative risk^a^ (95% CI)	Risk difference (95% CI)	P-value^b^	Pooled incidence (n = 118)
Baseline	5 (8.3%)	7 (12.1%)	1.40 (0.46 to 4.18)	3.7% (-7.2% to 14.7%)	0.70	12 (10.2%)
Peak of pain at first 24 h	26 (43.3%)	22 (38.0%)	0.87 (0.56 to 1.35)	5% (-12% to 23%)	0.55	28 (43.3%)
After 24h	10 (16.7%)	13 (22.4%)	1.34 (0.64 to 2.82)	-6% (-20% to 8%)	0.43	23 (17.0%)
After 72h	5 (8.3%)	4 (6.9%)	0.82 (0.23 to 2.93)	1.4% (-8% to 11%)	0.77	9 (8.3%)
After 7 days	1 (1.7%)	1 (1.8%)	1.05 (0.06 to 16.42)	-0.1% (-4.7% to 4.6%)	0.99	2 (1.7%)
Flare-up	2 (3.3%)	2 (3.4%)	1.07 (0.15 –to 7.34)	0.1% (-6% to 6%)	0.98	4 (3.3%)

HFLC,Hand File, Lateral Compaction technique; SFSC,Single-file and single-cone technique; CI,Confidence Interval; VRS, Verbal Rating Scale

^a^—Calculated using HFLC as control.

^b^—Chi square test.

In the intention to treat analyses, the SFSC group presented a mean PAI score of 2.07 (SD = 1,07) 12-months after treatment while the HFLC group presented mean PAI score of 2.17 (SD = 1,05). The confidence interval for the mean difference in PAI score and its corresponding confidence interval was -0.1 (-0.48 to 0.28). The periapical status of teeth in both groups was the following: Healed (40 teeth [66.7%] in the SFSC and 41 teeth [68.3%] in the HFLC group); Healing (9 teeth [15%] in the SFSC group and 11 teeth [18.3%] in the HFLC group); and Not Healed (11 teeth [18.3%] in the SFSC group and 12 teeth [20%] in the HFLC group). The periapical status of teeth at the 12-month follow-up was not related to the endodontic treatment technique used (Chi-square analyses, p = 0.85). The success rate was 0.67 (CI = 0.53 to 0.78) and 0.68 (CI = 0.55 to 0.80) for SFSC and HFLC groups, respectively. The pooled success rate was 0.68 (CI = 0.58 to 0.76).

Endodontic treatment using reciprocating single-file instrumentation and the single-cone filling technique simplified the treatment. However, the use of SFSC must be supported by clinical trials demonstrating the effectiveness of this simpler procedure. In the present pragmatic clinical trial, we tested the hypothesis that the simpler protocol combining reciprocating single-file instrumentation and a matching-taper single cone yields a rate of apical periodontitis healing equivalent to that observed with hand instrumentation followed by root canal filling using the lateral compaction technique. The findings of the present trial demonstrated that the difference in PAI changes between the treatments did not exceed the pre-established equivalence bound, thus, leading us to accept the hypothesis of the study.

Unlike explanatory trials, which are designed to evaluate any treatment under ideal experimental conditions, pragmatic clinical trials are performed in the routine of daily clinical practice under more real-life perspective [20]. Additionally, complex interventions, consisting of several interacting components or stages are usually assessed in pragmatic trials [21]. The endodontists who contributed to this study frequently use the lateral compaction technique to obturate root canals prepared with hand files in their practice in public services, and this protocol was defined as the control in the present trial. We also chose to use a different root filling technique for SFSC because the use of a single cone to fill the root canals (common for canals instrumented with NiTi rotary or reciprocating systems) increases the simplification of endodontic therapy.

An equivalence hypothesis test was chosen instead of the more usual two-sided superiority hypothesis to assess the success rate of apical periodontitis healing. Equivalence trials are designed to demonstrate that an experimental treatment results in a main outcome similar to the control’s, but its use must be indicated for other reasons (e.g. being less time-consuming). In addition, equivalence trials prevent the incorrect conclusion of non-significant p-values as the absence of an effect, which is common in superiority trials [22].

For the definition of success, a stricter criterion was used and only healed lesions (absence of apical radiolucency) were considered as such. In fact, a prior systematic review reported that the success criterion influences the results, and that using a stricter criterion results in a 10%-lower success rate than that observed when a loose criterion (any reduction in apical radiolucency) is used [23]. In the present study, the success rate observed after a 12-month follow-up (78% and 73% for SFSC and HFLC, respectively) would increase to over 95% (95.5% and 95.2% for SFSC and HFLC, respectively), which is consistent with the success rate observed in other clinical studies [13,23–24]. Our findings also demonstrated that the success rate was not affected by the demographic characteristics of the participants (e.g. age, race, gender), and the only clinical aspect observed at baseline affecting this outcome was the PAI. Teeth presenting lesions on PAI scores of 5 or 4 presented a reduction on odds of success by 94% and 91%, respectively, when compared to the lesions on a PAI score of 3 at baseline. In fact, larger apical lesions present the worst prognosis [24] and might take longer to completely heal [25].

We decided to report the per-protocol analyses as the main results because the results of the Little’s MCRA test indicated that the loss-to-follow-up in this study was entirely at random and, in this scenario, per-protocol analyses can be performed without introducing bias in the results. However, we also performed an ITT analysis, and it validated the per-protocol results. In both analyses, the confidence interval of the mean PAI difference did not exceed the 0.5-bound pre-established as the equivalency limit. Therefore, no significant differences between the two groups occurred regarding the periapical status distribution of teeth and the success rate of treatment. As expected, both protocols yielded lower PAI score reduction, reduced proportion of teeth presenting a “healed” periapical status, and lower success rate when compared to the per-protocol results.

The inclusion of different clinical settings is an important aspect of pragmatic trials since it improves the external validity of findings. In the present study, the participants were recruited in four clinical settings and treated by three clinicians, but these important variables were not included in the multivariate regression analysis. The absence of effect of these variables in the main outcome might be related to an unbalanced number of participants among the settings. In fact, two settings recruited few participants (26 and 10 for Estância and Capela, respectively), impairing that the operator and clinical settings would affect the main outcome. Therefore, the low number of participants in these two clinical settings was a limitation of this trial. However, we believe that this fact did not affect the results of the trial since similar success rates and a low PAI scores difference were observed between the treatments.

Further to apical periodontitis healing, the incidence and intensity of post-operatory pain and occurrence of flare-up were also assessed. Approximately 10% of the teeth evaluated presented some pain prior to the endodontic treatment, but the level of pain was low. The percentage of participants reporting any tooth pain increased to approximately 40% at first 24h following both protocols of endodontic treatment, and the incidence of pain gradually reduced over time to 2% after 7 days, corroborating prior studies [26]. The same behavior was observed for the intensity of pain, with no difference between the endodontic protocols. Different results were observed in a prior clinical trial that found a statistically lower level of postoperative pain when manual instrumentation was used, but the small difference between the treatments (around 0.3 at VAS) indicates a probable absence of clinical effect [27]. Regarding the occurrence of flare-up, only one case (3.3%) for each protocol was observed, and this incidence is lower than that (8.4%) reported in a prior meta-analysis [28]. However, differently from the present study, all types of teeth were included in that meta-analysis. In the present study, the final apical diameter was not standardized since a more pragmatic approach was used. Therefore, clinicians determine final apical diameter in daily routine clinical practice according to: 1) the anatomy of the tooth-receiving root canal treatment; and 2) the file system and instrumentation technique used. It is important to emphasize that, despite the statistical difference observed in the apical diameter, the small difference (0.6 and 0.5 mm for HFLC and SFSC, respectively) did not impact the outcomes evaluated. In fact, small differences in apical diameter seem to not affect the long-term prognoses of endodontic treatment [13].

It has been demonstrated that the root canal filling technique affects neither postoperative pain nor apical periodontitis healing [29,30]. Therefore, the ability of the protocol of instrumentation to reduce the microbial content in the root canal seems to be more important to achieve reduced postoperative pain and improved apical periodontitis healing. Indeed, prior ex-vivo evaluations demonstrated similar effects of hand files and NiTi single file applied in reciprocating motion to the microbial content in the root canals [31–33]. Additionally, filling the root canals with a matching-taper single cone seems to result in similar quality of root filing than that achieved using the lateral compaction technique [34]. It is important to emphasize that the pragmatic design of this study is a hindrance to defining if a factor has a higher impact on the outcomes. However, despite the limitations of pragmatic trials, this design facilitates the generalization of the findings for daily clinical practice in similar conditions.

## Conclusions

Within the limitations of this study, it is possible to conclude that a simplified endodontic treatment combining instrumentation with a single file and root canal filling using a matching-taper single cone was as effective on apical periodontitis healing and postoperative pain as manual instrumentation followed by the lateral compaction obturation technique.

## Supporting information

S1 Checklist CONSORT 2010 checklist of information to include when reporting a randomised trial*. (DOC)

S1 Dataset (XLSX)

S1 Protocol (DOCX)

S2 Protocol (DOCX)
