# Correction: Apical periodontitis healing and postoperative pain following endodontic treatment with a reciprocating single-file, single-cone approach: A randomized controlled pragmatic clinical trial

**DOI:** 10.1371/journal.pone.0230511

**Published:** 2020-03-11

**Authors:** Fabricio Eneas Diniz de-Figueiredo, Laila Fernandes Lima, Giana Silveira Lima, Ludmila Smith Oliveira, Maria Amália Ribeiro, Manoel Brito-Junior, Marcos Brito Correa, Manoel Sousa-Neto, André Luis Faria e Silva

The eighth author’s initials are indexed incorrectly in PubMed. The correct initials are: Sousa-Neto MD.
