# Supplementary material for: Apical periodontitis healing and postoperative pain following endodontic treatment with a reciprocating single-file, single-cone approach: A randomized controlled pragmatic clinical trial
Source: PLoS One. 2020 Feb 3;15(2):e0227347. doi: 10.1371/journal.pone.0227347 (PMC6996828; doi:10.1371/journal.pone.0227347)
Supplement: S1 Protocol — (DOCX) [file pone.0227347.s003.docx]

**Projeto de pesquisa**

**Eficácia do sistema Reciproc no tratamento endodôntico de dentes anteriores com lesão periapical: ensaio clínico pragmático randomizado controlado**

**Proponente:**

Doutorando Fabricio Eneas Diniz de Figueiredo

Programa de Pós-Graduação em Ciências da Saúde

Universidade Federal de Sergipe

**Orientador:**

Prof. Dr. André Luis Faria e Silva

Departamento de Odontologia

Campus da Saúde / Centro de Ciências Biológicas e da Saúde

Universidade Federal de Sergipe

Aracaju

Junho, 2015

**RESUMO**

A técnica de instrumentação de canais radiculares com único instrumento reciprocante surgiu como uma tentativa de superar as limitações apresentadas pelas técnicas manuais e de rotação continua. Apesar da facilidade técnica, poucos estudos avaliaram a efetividade do uso de instrumentação reciprocante na prática clínica. Assim, o objetivo desta proposta será avaliar o desempenho clínico da instrumentação reciprocante e obturação com cone único do sistema Reciproc®, no que concerne ao índice de sucesso do tratamento endodôntico e à incidência de dor pós-operatória, através de um ensaio clínico randomizado controlado. Este será realizado seguindo as recomendações do *Consort*, usando controle ativo – instrumentação manual pela técnica coroa-ápice e obturação pela técnica da condensação lateral. Pacientes que apresentem dentes anteriores com quadro clínico de necrose pulpar e evidência radiográfica de periodontite apical serão randomizados para tratamento endodôntico com limas do sistema Reciproc® ou para tratamento com limas manuais. O paciente será questionado qual seu nível de dor percebida antes de iniciar o tratamento e 24 horas, 72 horas e 7 dias após sua realização; os escores serão submetidos ao teste de Wilcoxon para comparação das técnicas endodônticas. Doze meses e um ano após o tratamento, os pacientes serão convocados para nova avaliação clinica e radiográfica, na qual serão verificadas alterações no índice PAI com relação ao registrado no *baseline* e sinais ou sintomas clínicos de periodontite periapical. Os dados relativos ao índice PAI serão submetidos ao teste de Wilcoxon para comparação entre as técnicas e ao teste de Man-Whitney para comparar os scores observados na avaliação de 1 ano. O sucesso de cada tratamento será caracterizado se o dente apresentar índice PAI igual ou inferior a 2 e na ausência de sintomatologia clínica. As percentagens de sucesso de cada tratamento e o risco relativo do tratamento experimental em relação ao controle serão calculados. Além disso, as percentagens de sucesso de cada tratamento serão comparadas pelo teste exato de Fisher. Para todas as análises, será utilizado nível de significância estatística de 95%.

**Palavras-chave:** Periodontite Periapical; Preparo de Canal Radicular; Ensaio Clínico**ABSTRACT**

Single file canal preparation techniques emerged in an attempt to overcome drawbacks related to manual and continuous rotary techniques. It has shown promising results in in-vitro studies, but its efficacy has not yet been tested in clinical studies. Hence, this proposal seeks to investigate, through an equivalency randomized controlled clinical trial, the efficacy of the Reciproc single file, single cone system, regarding its success rate, incidence rate of post-operative pain and PAI score changes. This trial will follow the Consort guidelines, having a parallel design and using an active treatment as control – the crown-down instrumentation technique and the lateral condensation obturation technique. Patients in need of endodontic treatment for anterior teeth due to pulp necrosis and with radiographic evidence of apical periodontitis will be randomized to receive endodontic treatment either by the Reciproc single file, single cone system or by the crown-down manual technique with stainless steel files. Patients will be questioned about their pain levels at baseline and then 24 hours, 72 hours and 7 days after treatment is performed. The Wilcoxon test will be used to compare treatment pain scores. Patients will be called for one- year follow-up visits in which PAI score changes and clinical signs of periapical pathosis will be ascertained. Data related to PAI scores will be submitted to the Wilcoxon test to compare treatment outcomes and to the Man-Whitney test to compare one-year follow-up scores within each treatment group. Each individual treatment will be classified as “success” if their PAI scores are 2 or lower and they show no clinical signs or symptoms of periapical disease. Success rates of each treatment and the relative risk of experimental group in relation to control will be determined. In addition, Fishers exact test will be used to compare treatment groups’ success rates. For all analyses, a 95% significance level will be used.

**Key Words:** Periapical Periodontitis; Root Canal therapy; Randomized Controlled Clinical Trial

# 1. Introdução

## 1.1 Estabelecimento do problema

A terapia endodôntica tem como um de seus objetivos tratar a inflamação dos tecidos periapicais, causada pela presença de microrganismos no sistema de canais radiculares (SCR) (1). Para alcançá-lo, é necessário que esses microrganismos sejam eliminados ou removidos, o que, até o momento, é obtido por meio da limpeza e modelagem do SCR com o uso de limas endodônticas, auxiliadas pelo uso concomitante de uma solução irrigadora com propriedades antimicrobianas (2). Tradicionalmente, os procedimentos de limpeza e modelagem são realizados com limas endodônticas de aço inoxidável acionadas manualmente. Porém, por apresentarem baixa flexibilidade, a instrumentação de canais curvos com essas limas pode levar a deformação da anatomia do canal (3) o que é indesejável. Para minimizar este problema, surgiu a técnica de instrumentação rotatória, na qual limas de níquel titânio (NiTi) – que apresentam super elasticidade - são acionadas a motor em movimento de rotação contínua. Com esta técnica, houve a diminuição do tempo de trabalho e do estresse do operador, permitindo que canais radiculares pudessem ser preparados com maior eficiência e segurança. No entanto, as principais desvantagens destas técnicas rotatórias estão relacionadas com a possibilidade de fratura do instrumento e a necessidade de empregar uma série de instrumentos para a completa limpeza e modelagem do SCR (4,5).

Como tentativa de simplificar a instrumentação endodôntica, surgiu a filosofia do tratamento endodôntico “lima única, cone único”. Nesta filosofia, uma única lima de NiTi é utilizada para realizar todas as etapas da instrumentação endodôntica. O movimento de rotação contínua foi substituído pelo movimento reciprocante, visando diminuir o risco de fratura (6). No entanto, apesar da facilidade da técnica, há escassas evidências acerca da efetividade clínica do uso da técnica de “lima única, cone único”. Pelo contrário, algumas características observadas nos estudos laboratoriais sugerem potenciais problemas com seu uso clínico. Em função da instrumentação muito rápida, a substância irrigadora auxiliar pode não atuar por tempo suficiente para que uma adequada desinfecção do SCR, o que pode comprometer o tratamento. Além disto, alguns estudos demonstraram que a extrusão de debris (7, 8) pelo forame apical é maior que nas outras técnicas, o que pode elevar a incidência de dor pós-operatória.

## 1.2 Estado da arte

O primeiro sistema de instrumentação com lima única – o Reciproc - foi descrito por Yared (9,10). De acordo com o autor, suas principais vantagens são a facilidade e simplicidade de execução, com menor risco de fratura da lima e que elimina a contaminação cruzada. Vários estudos laboratoriais confirmaram maior resistência de instrumentos reciprocantes à fadiga cíclica e o menor tempo de execução da técnica (11,12,13). Porém, também em estudos laboratoriais, além de não ter se mostrado superior às outras técnicas de instrumentação no que concerne a eficácia antibacteriana (14), foi observado que seu uso resulta em uma maior extrusão de debris (7) pelo forame apical, o que pode resultar em uma maior incidência de dor pós-operatória; fato este já demostrado clinicamente (15,16). No entanto, esta maior extrusão com o uso da lima única não é consenso entre os pesquisadores (17). Clinicamente, desconhecemos estudos que comparam o desempenho da instrumentação com lima única com outras técnicas de instrumentação. Na literatura, encontramos um estudo que relatou índice de sucesso de 93% com o uso de instrumentos reciprocantes, em tratamento de dentes com periodontite apical, após três anos da execução do tratamento. No entanto, além de não ser comparativo, este estudo foi realizado pelo próprio criador do sistema, em sua clínica particular (18).

## 1.3 Identificação da proposta

Esta proposta objetiva por meio de ensaio clínico randomizado controlado, avaliar o desempenho clínico da instrumentação de canais radiculares com lima única do sistema Reciproc, no que concerne a cura de lesões periapicais e à incidência de dor pós-operatória.

**2. Justificativa**

Diminuir o tempo operatório do tratamento endodôntico pode auxiliar na redução do seu custo, aumentando a produtividade de clínicas publicas e privadas; além de tornar o tratamento mais ergonômico para o dentista e menos desconfortável para o paciente. No entanto, essa redução não deve comprometer os resultados imediatos e em longo prazo do tratamento. A filosofia de instrumentação de canais radiculares com lima única em movimento reciprocante surgiu como uma tentativa de simplificar e reduzir o tempo operatório da terapia endodôntica. Um desempenho clínico desta nova técnica similar à executada tradicionalmente, pode favorecer, principalmente, o atendimento público com redução de custos e aumento de produtividade. Portanto, investigar o desempenho clínico dessa nova filosofia de instrumentação de canais radiculares é importante para que gestores de clínicas públicas e privadas possam planejar melhor o atendimento durante a prática endodôntica

**3.Objetivos**

## 3.1- Geral

Avaliar a eficácia clinica do sistema de instrumentação com lima única sistema Reciproc em tratamento endodôntico de dentes unirradiculares apresentando periodontite apical.

## 3.2. – Específicos

- Comparar mudanças no índice PAI entre a instrumentação manual e a instrumentação reciprocante;
- Comparar o índice de sucesso clínico entre a instrumentação manual e a instrumentação reciprocante em dentes anteriores;
- Comparar a incidência de dor pós-operatória entre os dois tipos de instrumentação.
- Comparar a incidência de efeitos adversos (abscessos fênix) entre os dois tipos de instrumentação.

#

# 4. Metas

- Contribuir com o conhecimento cientifico relacionado à instrumentação de canais radiculares com a técnica de lima única e obturação pelo cone único;
- Contribuir na melhoria dos serviços públicos de endodontia ofertados através dos centros de Especialidades Odontológicas.
- Aprimorar a área de ensaios clínicos em Odontologia, no Programa de Pós-graduação em Ciências da Saúde da Universidade Federal de Sergipe, considerando que estes estão no topo da evidência científica primária para o estabelecimento da Odontologia baseadas em evidências na prática clínica;
- Constituir parcerias com outras instituições para delineamento execução de ensaios clínicos randomizados multicêntricos;
- Melhorar a qualidade de formação dos discentes vinculados ao Programa de Pós-graduação em Ciências da Saúde da Universidade Federal de Sergipe;
- Possibilitar a obtenção de uma tese de doutorado e de dois artigos publicados em periódicos com fator de impacto superior a 2.

# 5. Metodologia

## 5.1 Desenho experimental

Este projeto será composto por um ensaio clínico pragmágtico randomizado controlado, que será conduzido seguindo as recomendações do Consort (<http://www.consort-statement.org/>). Trata-se de um estudo intervencionista, *paralelo*, com taxa de alocação de 1:1, com uso de controle ativo (instrumentação manual) - e tendo de como desfecho principal mudanças no índice periapical *(PAI)* (19) 12 meses após realização do tratamento. O pico de dor nas primeiras 24 horas e dor pós-operatória 24 e 48 horas e 7dias após o procedimento serão os desfechos secundários.

O ensaio será baseado na seguinte questão PICO:

P (*Population* – população do estudo): pacientes adultos acima de 18 anos, que apresentem dentes anteriores com quadro clínico de necrose pulpar e evidência radiográfica de periodontite apical (tamanho mínimo > 2mm x 2mm), encaminhados aos serviços de endodontia: do Centro de Especialidades Odontológicas da prefeitura municipal de Nossa Senhora do Socorro, SE,; dos Centros de especialidades Odontológicas administrados pela Fundação Estadual de Saúde de Sergipe – FUNESA – localizados nas cidades de São Cristóvão, Laranjeiras e Capela.

I: (*Intervetion* – intervenção experimental): Terapia endodôntica realizada com a técnica de instrumentação reciprocante (Sistema Reciproc) e técnica de obturação com cone único (Técnica de cone único, lima única)

C: (*Control*– intervenção controle): Terapia endodôntica realizada com a técnica de instrumentação manual e técnica de condensação lateral para a obturação.

O: (*Outcomes* – resultado, ou desfecho primário): Índice de sucesso da terapia endodôntica após 1 ano medido por meio da mudança no índice PAI.

Portanto, a pergunta da questão é a seguinte: “Para pacientes que apresentam dentes anteriores com quadro clinico de necrose pulpar e periodontite apical crônica, existe diferença nos resultados do tratamento endodôntico com instrumentação pela técnica manual coroa-ápice ou com sistema reciprocante, no que concerne o índice de sucesso radiográfico, no índice de sucesso clinico, a incidência de dor pós-operatória, a incidência de efeitos colaterais – abscesso fênix - e na qualidade de vida?”. A hipótese principal a ser testada é a que o uso de lima e cone único resultará em resultados similares (equivalência) à da instrumentação manual seguida pela técnica da condensação lateral, em relação ao índice de sucesso radiográfico (redução no índice PAI). Além dessa, também será testada a hipótese de que não há diferença entre as duas técnicas de instrumentação no que concerne à incidência de dor pós-operatória à incidência de efeitos colaterais (abscesso fênix).

## 5.2 Método

### 5.2.1 Cálculo amostral

O cálculo do tamanho amostral foi feito para avaliar a diferença média nos escores PAI entre as duas modalidades de tratamento 12 meses após sua realização. Assim, para um desfecho contínuo, considerando similaridade entre os tratamentos (limite de equivalência de – 0,5 a 0,5 pontos na escala PAI), utilizou-se um desvio padrão de 0,73 (condição com os melhores resultados do estudo de Saini e Tewari et al. (2012), que foi alcançado com o alargamento 3 vezes maior que a primeira lima a alcançar o comprimento de trabalho de forma justa), erro tipo I de 5% e poder de teste de 90%. Ao resultado do cálculo, aumentou-se a amostra em 20% para compensar possíveis perdas de seguimento do período de avaliação. Desta forma, chegou-se ao resultado final de 60 dentes por condição experimental. O cálculo foi realizado no seguinte sítio: <https://www.sealedenvelope.com/power/continous-equivalence/>

### 5.2.2 Seleção da amostra

Para definição da amostra serão seguidos os seguintes critérios:

**Critérios de inclusão**

- Qualquer indivíduo que apresente dentes anteriores com quadro clínico de necrose pulpar e evidência radiográfica de periodontite apical, apresentando lesão superior a 2 mm em diâmetro;
- Estar de acordo em participar e assinar o termo de consentimento livre e esclarecido.

**Critérios de não inclusão**

- Dentes com grande destruição coronária, necessitando de reabilitação protética;
- Apresentar alguma condição clínica geral e/ ou oral preexistente que coloque o indivíduo em risco durante o estudo;
- Estar grávida ou lactante;
- Paciente com doença periodontal generalizada ou acometendo o dente que necessita de tratamento endodôntico;
- Reação alérgica as substâncias químicas utilizadas no tratamento endodôntico;
- Dentes que não apresentem a formação radicular completa – rizogênese incompleta.
- Dentes que apresentem evidência radiográfica de reabsorção radicular interna ou externa;
- Dentes cujo canal radicular esteja calcificado.
- Dentes submetidos a tratamento endodôntico prévio.
- Histórico de uso de antibiótico previamente ao tratamento; ou necessidade de profilaxia antibiótica (ex. endocardite bacteriana).

**Critérios de exclusão**

- Dentes que apresentem tecido pulpar vital, mesmo que radiograficamente haja presença de imagem radiolúcida associada ao periápice;
- Dentes que, durante o tratamento endodôntico, apresentarem sinais de fratura radicular.
- Quando, durante o procedimento endodôntico, ocorrerem acidentes e complicações, especificamente perfurações radiculares e separação de instrumento dentro do SCR.
- Pacientes que não retornarem às consultas de reavaliação.

Após a seleção dos pacientes, será realizada a abertura de prontuário, o mesmo utilizado no serviço de origem do paciente. Em seguida, o paciente deverá assinar o termo de consentimento livre e esclarecido (Anexo I).

### 5.2.3 Avaliação inicial (baseline)

Para avaliar a elegibilidade dos pacientes, a condição pulpar e periapical serão avaliadas através de testes térmicos – frio e calor – de sensibilidade pulpar e de radiografias periapicais realizadas pela técnica do paralelismo. Também será realizada uma avaliação clínica (inspeção visual, palpação no fundo de sulco, percussão vertical e horizontal) para registrar presença ou ausência de edema, fístula, mobilidade anormal e dor.

Confirmado o quadro de necrose pulpar e periodontite apical, os demais critérios de elegibilidade, e caso o paciente aceite participar do estudo e assine o termo de consentimento, o valor inicial do índice PAI de cada participante será registrado. Para fazê-lo, as radiografias serão posicionadas em negatoscópio e coberta com papel fotográfico preto com uma abertura de 10 mm, de tal forma que apenas a região periradicular esteja visível (19). Após inspeção da área com lupa de magnificação 5X, será registrado o valor conforme a tabela 1.

Tabela 1. Índice PAI (19).

| VALOR PAI | DESCRICAO DOS ACHADOS RADIOGRÁFICOS |
| --- | --- |
| 1 | Estruturas Periapicais Normais |
| 2 | Pequenas mudanças na estrutura óssea |
| 3 | Mudanças na estrutura óssea com perda mineral |
| 4 | Periodontite com área radiolúcida bem delimitada |
| 5 | Periodontite severa |

Caso o paciente afirme estar sentido dor relacionado ao dente no momento da avaliação inicial, seu nível também será utilizando escala verbal descrita na tabela 2. Se o paciente não se queixar de dor, será registrado o valor 0, que corresponde à ausência de dor.

Tabela 2. Escala de dor pós-operatória (21).

| Escala de dor pós-operatória, em 5 níveis; Valores dados aos pacientes | | |
| --- | --- | --- |
| 0 | Ausência de dor | O paciente se sente bem |
| 1 | Dor Leve | Se distraído, o paciente não percebe a dor |
| 2 | Dor Moderada | O paciente sente dor moderada, mesmo quando concentrado em outra atividade |
| 3 | Dor Severa | O paciente não se sente bem, mas consegue realizar suas tarefas diárias |
| 4 | Dor Muito Severa | O paciente é obrigado a deixar de lado suas tarefas diárias |
| 5 | Dor Insuportável | O paciente é incapaz de realizar qualquer tarefa e precisa se deitar para repouso |

### 5.2.4 Aleatorização

Para definição do protocolo de tratamento endodôntico a ser realizado, a lista de randomização será criada utilizando a página www.sealedenvelope.com. Para isto será utilizado um iniciador (seed) em 1, dois grupos, para uma lista de extensão de 120, em blocos de 2, sendo este valor definido pelos locais onde serão realizadas a pesquisa. A lista será gerada por um operador que não participará das intervenções e/ou avaliações. Este produzirá envelopes lacrados numerados de 1 a 120, contendo em seu interior a técnica de instrumentação e obturação endodôntica a ser utilizada naquele paciente. O número do paciente será dado em ordem de triagem. O operador encarregado de realizar o tratamento endodôntico apenas abrirá o envelope no momento de iniciar a instrumentação endodôntica.

### 5.2.5 Intervenção

Os pacientes alocados para a intervenção serão submetidos a tratamento endodôntico pela técnica da lima única e cone único, utilizando o sistema Reciproc. Já os pacientes alocados para o controle serão submetidos a tratamento endodôntico com limas manuais de aço inoxidável e com obturação do canal radicular pela técnica da condensação lateral.

### 5.2.6 Protocolo de tratamento

Na sessão em que será realizado o tratamento endodôntico, antes de iniciar o procedimento, a radiografia de diagnóstico do dente será utilizada para obtenção do comprimento de trabalho provisório (CTP), que será obtido subtraindo-se 1 mm do comprimento do dente na radiografia. Após essa determinação, a proteção individual do profissional será realizada com uso de gorro, óculos de proteção, máscara, luvas, avental; e do paciente, com óculos de proteção. O paciente será orientado a fazer um bochecho de 1 minuto com solução de clorexidina a 0,2%. O dente será anestesiado com solução de lidocaína 2% e epinefrina 1:100.000 (DFL, RJ, Brasil) pela técnica infiltrava. O isolamento absoluto do dente será realizado com arco de ostby, lençol de borracha e grampos endodônticos para isolamento. Em seguida, será realizada a remoção de todo tecido cariado com broca esférica em baixa rotação. O acesso à câmara pulpar será feito através de pontas esféricas diamantadas números 1011, 1012 ou 1013 ou brocas esféricas carbide para alta rotação números 2 ou 3 acionadas com caneta de alta rotação, levando em consideração todos os princípios que norteiam a abertura coronária.

Nos pacientes randomizados para o grupo controle, o tratamento endodôntico será realizado pela técnica coroa-ápice, utilizando-se brocas de Gattes-Glidden (Dentsply Maillefer, Ballaigues, Suiça) para o preparo do terço cervical e limas manuais de aço inox (Dentsply Maillefer, Ballaigues, Suiça) para preparo dos terços médio e apical; e a obturação endodôntica realizada pela técnica da condensação lateral, de acordo com a seguinte sequência:

1) Exploração inicial do canal radicular com limas número 10 e 15 até o CTP;

2) Preparo dos terços cervical médio: Broca 3083 (KG Sorensen, Barueri, SP, Brasil) para remoção do ombro de dentina na face lingual do canal; Brocas de Gates -Glidden números 3, 2 e 1 até o limite de dois terços da extensão do canal;

3) Odontometria: Será utilizado um localizador foraminal eletrônico, acoplado ao motor elétrico RomiApex A-15 (Romidan, Kiryat Ono, Israel), para determinação do comprimento de trabalho (CT). Este será estabelecido subtraindo-se 1mm do comprimento no qual o localizador indicar como sendo a localização do forame apical;

4) Preparo do terço apical: Limas tipo K de aço inoxidável (Dentsply Maillefer, Ballaigues, Suiça) serão empregadas na profundidade do comprimento de trabalho determinado na Odontometria, em ordem crescente de diâmetro, sendo o alargamento final determinado pela anatomia da região.

6) Remoção da camada hibrida com uso de solução de EDTA 17% agitado durante 03 minutos dentro do canal radicular.

7) Irrigação final com 5ml de hipoclorito de sódio 2,5%;

8) Prova do cone: Um cone de guta percha correspondente a última lima utilizada no preparo apical, será introduzido no canal para verificar se ele alcança o CT e se seu travamento está adequado (quando há resistência ao deslocamento coronário). Em seguida, será realizada uma radiografia com o cone posicionado dentro do canal para avaliar se ele alcança o limite apical de trabalho.

9) Secagem do canal com pontas de papel absorvente (Dentsply Maillefer, Ballaigues, Suiça) correspondente a lima utilizada para realizar a instrumentação;

10) O cone selecionado será untado com cimento endodôntico Sealer AH Plus (Dentsply Maillefer, Ballaigues, Suiça) manipulado de acordo com as recomendações do fabricante, e introduzido no canal até o comprimento de trabalho. Cones secundários (Dentsply Maillefer, Ballaigues, Suiça) serão introduzidos com auxilio de espaçador digital (Dentsply Maillefer, Ballaigues, Suiça) para a realização da condensação lateral, até o momento em que este não penetrar mais que 2 mm do terço cervical. O excesso será cortado com calcador de Paiva aquecido e em seguida, a guta percha ainda aquecida, realizar-se-á a condensação vertical da guta percha, deixando o material obturador 2 mm do colo anatômico.

Já nos pacientes randomizados para o grupo de intervenção, o tratamento endodôntico será realizado pela técnica descrita por Yared (8) e obturados pela técnica do cone único de acordo com a seguinte sequência:

1) Seleção do instrumento a ser utilizado: Se, na avaliação radiográfica, a imagem do SCR for visível desde a câmara pulpar até o ápice, um instrumento manual de numero 30 deve ser inserido passivamente até o CTP do dente. Caso está lima alcance passivamente o CTP o canal é considerado amplo e um instrumento R50 (VDW GmbH, Munique, Alemanha) deve ser selecionado. Caso a lima não alcance o CTP, uma lima numero 20 deve ser introduzida no canal. Caso ela alcance de forma passiva o CTP, o canal é considerado médio e uma lima R40 (VDW GmbH, Munique, Alemanha) deve ser selecionado. Caso a lima 20 não alcance o CTP, o canal é considerado atrésico e uma lima R20 (VDW GmbH, Munique, Alemanha) é selecionada.

2) Preparo dos terços cervical e médio: O stop de silicone da lima selecionada será posicionado em aproximadamente 2/3 do CTP e introduzido no canal, acionado a motor em programação especifica, com movimento de “entrada e saída”, sem que o mesmo seja completamente retirado do canal. Após três ciclos de entrada e saída, ou ao encontrar algum tipo de resistência, o instrumento deve ser removido e o canal abundantemente lavado. Essa sequência é repetida até que o instrumento alcance os 2/3 previamente estabelecidos.

3) Odontometria será realizada por meio de localizador foraminal eletrônico, acoplado ao motor elétrico RomiApex A-15 (Romidan, Kiryat Ono, Israel), para determinação do comprimento de trabalho (CT). Este será estabelecido subtraindo-se 1 mm do comprimento no qual o localizador indicar como sendo a localização do forame apical;

4) Preparo do terço apical será feito utilizando a mesma lima cinemática descrita anteriormente.

5) Remoção da camada hibrida com uso de solução de EDTA 17% agitado durante 03 minutos dentro do canal radicular.

6) Irrigação final com 5ml de hipoclorito de sódio 2,5%;

7) Prova do cone: Um cone de guta percha (VDW GmbH, Munique, Alemanha), correspondente a lima utilizada para instrumentação do SCR, será introduzido no canal para verificar se ele alcança o CT e se seu travamento está adequado (quando há resistência ao deslocamento coronário). Em seguida, será realizada uma radiografia com o cone posicionado dentro do canal para avaliar se ele alcança o limite apical de trabalho.

8) Secagem do canal com pontas de papel absorvente (VDW GmbH, Munique, Alemanha) correspondente a lima utilizada para realizar a instrumentação;

9) O cone selecionado será untado com cimento endodôntico AH Plus (Dentsply, New York, USA), manipulado de acordo com as recomendações do fabricante, e introduzido no canal até o comprimento de trabalho. Em seguida, o excesso de material será removido com uso de condensador de Paiva (SS White Duflex, Rio de Janeiro, Brasil) numero 2 previamente aquecido. Após a remoção do excesso e com a guta percha ainda plastificada, realizar-se-á condensação vertical com condensador de Paiva a frio, deixando o material obturador 2 mm do colo anatômico.

Em ambos os grupos, será utilizado solução de hipoclorito de sódio 2,5%, tanto na irrigação dos canais radiculares quanto como auxiliar na instrumentação. A cada troca de lima, o canal será irrigado com 10 ml da solução e uma lima 10 ou 15 será utilizada em toda a extensão do dente para manter o SCR e o forame apical patentes; livres de obstruções e raspas de dentina. Cada lima Reciproc será utilizada somente uma vez como recomendado pelo fabricante.

Os dentes serão restaurados provisoriamente com cimento de ionômero de vidro e os pacientes orientados a retornarem após uma semana para a realização da restauração definitiva com resina composta.

A fim de padronizar as radiografias, serão utilizados filmes radiográficas periapicais Kodak *UltraSpeed* nº 2, sensibilidade D (Kodak São Paulo, Brasil) posicionadas com auxílio de posicionador radiográfico (Rinn XCP, Dentsply EUA). Para que as incidências radiográficas da avaliação inicial e de proservação de um mesmo paciente sejam realizadas na mesma posição, o posicionador radiográfico será estabilizado com material de impressão (silicone de condensação) Optosil Comfort (Heraus Kulzer – Alemanha), que será colocado sobre o bordo incisal dos dentes radiografados. Este molde será confeccionado para realização da radiografia de diagnóstico, e utilizado para realização da radiografia final do tratamento endodôntico, e das radiografias de proservação de 1 ano. Após cada uso será lavado em água corrente, desinfetado com solução de hipoclorito de sódio a 1% durante 10 minutos e armazenado em recipiente individual, identificado com o nome do paciente e mantido sob refrigeração para evitar distorções (22).

Será utilizado aparelho de raios X Spectro II (Dabi-Atlante, Brasil), com quilovoltagem pico de 67 kVp e miliamperagem fixa de 8 mA. O tempo de exposição utilizado será o recomendado pelo fabricante. O processamento radiográfico será realizado de forma manual, pelo método temperatura / tempo, com soluções novas preparadas de acordo com as instruções do fabricante (Kodak, São Paulo, Brasil).

### 5.2.8 Avaliações

Trinta radiografias periapicais de tratamentos endodônticos que não fazem parte deste estudo serão usadas nos procedimentos de calibração de dois avaliadores independentes e cegos. A avaliação destas radiografias será realizada até se atingir uma concordância

Doze meses após a realização do tratamento, o paciente será contatado e solicitado a comparecer para uma consulta de proservação. Nesta consulta, será registrado o segundo valor do índice PAI conforme descrito anteriormente. A média de cada grupo será calculada, comparada com a média da avaliação inicial e a redução média de cada grupo calculada. Além disso, os dados relativos ao índice PAI serão convertidos em uma escala de dados categóricos nominais, na qual dentes com valor PAI ≤ 2 serão considerados como curados, enquanto que dentes com valor ≥ 3 serão considerados como não curados. Também será realizada uma avaliação clinica para determinar o índice de sucesso clinico. Ausência de dor e edema à palpação, ausência e/ou regressão de fistula e o dente funcional serão considerados sucesso clínico, enquanto que, na presença de um ou mais desses sinais e sintomas, o tratamento será considerado como fracasso clínico. Para determinar o índice de sucesso dos tratamentos realizados, o dente precisa apresentar sucesso clínico e ser classificado como curado na avaliação radiográfica.

Os casos em que ocorrer dor pós operatória serão registrados tanto por meio de uma escala visual analógica (EVA) quanto por meio da escala verbal. Para o registro na EVA, o paciente vai indicar seu nível de dor com uma marcação (com uma caneta) em cima de uma linha de 10 cm entre dois extremos (um indicando ausência de dor e o outro indicando dor insuportável). A distância entre a marcação do paciente e o extremo da linha que indica ausência de dor será mensurara e registrada. Já para o registro na escala verbal de dor, será utilizada os escores descritos na tabela 2. A escala verbal também será utilizada para avaliar a incidência de dor pós-operatória (valores diferentes de 0). A ocorrência do efeito adverso “abscesso fênix” será registrado nos casos em que o paciente apresente dor severa e edema logo após o tratamento, e consequentemente, necessitar de uma consulta de urgência não agendada. Além do registro do efeito colateral, estes casos também serão registrados como insucesso clínico, e a participação deles no estudo será encerrada.

### 5.2.8 Cegamento

Em virtude das diferenças perceptíveis nas técnicas, não será possível realizar o cegamento do operador e do paciente. Assim, apenas o avaliador dos desfechos não saberá o tratamento recebido pelo paciente, constituindo o estudo em simples cego.

## 5.3 Análise dos dados

As características demográficas dos participantes serão categorizadas, sendo então calculadas frequências relativas e absolutas para cada categoria. Diferenças entre os tratamentos (intervenção e controle) em relação a essas características serão analisadas por meio do teste do Qui-quadrado, enquanto diferenças em relação ao diâmetro apical final, tempo de proservação, idade e PAI serão analisadas pelo teste de Mann-Whitney.

Para todos os dados provenientes de EVA, o teste de Shapiro-Wilk será utilizado para averiguar a ocorrência de distribuição normal dos mesmos, e possíveis diferenças entre os tratamentos serão investigadas por meio do teste T. Já para os dados provenientes da escala verbal, diferenças entre as técnicas endodônticas serão averiguadas por meio do teste de Wilcoxon. Além da comparação entre os escores, o risco à sensibilidade também será avaliado. Para isto, inicialmente, será determinado o risco absoluto a sensibilidade dental para cada técnica endodôntica, em cada tempo de avaliação, através da razão entre casos que apresentaram escore diferente de zero (alguma sensibilidade) e o total de casos. As diferenças de risco serão calculadas, assim como os intervalos de confiança correspondentes. Em cada tempo, os riscos absolutos dos tratamentos serão comparados através do teste Exato de Fisher.

Para testar a hipótese de equivalência entre as duas técnicas endodônticas em relação a mudanças nos escores PAI, será utilizado o procedimento estatístico que inclui dois testes uni-caudais (“*two one sided tests*” – TOST) para dados não paramétricos. (LAKENS, 2017). Um modelo de regressão logística será criado para investigar a relação das características clínicas e demográficas dos pacientes com a taxa de sucesso do tratamento passados 12 meses de sua realização. Inicialmente, regressão logística univariada foi utilizada para explorar a relação de cada característica clínico-demográfica individualmente com o sucesso do tratamento. Características que apresentarem valor de p < 0,1 na análise univariada serão selecionadas para fazerem parte do modelo final com análise multivariada. A relação entre o sucesso do tratamento e as características serão incluídas no modelo final expressa em razão de chances.

# 6. Contribuições científicas da proposta

Esta proposta poderá ajudar a contribuir com o conhecimento dos sistemas de instrumentação endodôntica, o que ajudará a melhorar o ensino, aprendizagem e pratica clínica da Endodontia. Além disso, poderá contribuir no planejamento e gerenciamento de serviços públicos e privados de Endodontia. Considerando que os ensaios clínicos randomizados controlados são a melhor evidência científica para aplicações clínicas, pela maior capacidade de controlar fatores de confusão, espera-se que os resultados desta proposta auxiliem na prática da odontologia baseada em evidências científicas.

**Referências**

1.Trope M. The vital tooth – its importance in the study and practice of endodontics. *Endo Topics* 2003;5:1 .

2. Siqueira Jr JF, Roças IN. Microbiology and treatment of endodontic infections. In: Hargreaves KM, Cohen S. Pathways of the pulp. 10 Ed. St Louis: Mosby Elsevier, 2001;559-600

3. Pereira HSC, Silva EJNL, Coutinho-Filho TS. Movimento Reciprocante em Endodontia: Revisão de Literatura. *Rev bras odontol* 2012;69(2):246-249.

4. Yared G. Canal preparation with nickel-titanium or stainless steel instruments without the risk of instrument fracture: prelimirary observations. *Restor Dent Endod* 2014.

5. Peters OA. Current challenges and concepts in the preparation of root canal systems: A review. *J Endod* 2004; 30(8):559-567.

6.Çapar I, Arslan H. A review of instrumentation kinematics of engine-driven nickel-titanium instruments.’ Accepeted article’, doi:10111/iej.12432

7. Burklein S, Schafer E. Apically extruded debris with reciprocating single-file and full-sequence rotary instrumentation systems. *J Endod* 2012; 38(6): 850-852.

8. Nayak G, Singh I, Shetty S, Dahiya S. Evaluation of apical extrusion of debris and irrigant using two new reciprocating and one continues rotation single single file system. *J Dent (Tehran)* 2014; 11(3):302-309.

9. Yared G. Canal preparation with only one Ni-Ti rotary instrument: preliminar observations. *Int Endod J* 2008; 41:339-344.

10. Yared G. Canal Preparation with only one Reciprocating Instrument without prior hand filing. A new concept. 2001. Disponível em: <http://www.vdwreciproc.de/images/stories/pdf/GY_Artikel_en_WEB.pdf>.

11. Frota MF et al. Comparison of cyclic fatigue and torsional resistance in reciprocating single-file systems and continuous rotary instrumentation systems. *J Oral Sc* 2014; 56(4):269-275.

12.Kiefner P, Ban M, De-Deus G. Is the reciprocating movement per se able to improve the cyclic fatigue resistance of instruments? *Int Endod J* 2014; 47(5):430-436.

13. Katge F, Patil D, Poojari M, Pimpale J, Shitoot A, Rusawat B. Comparison of instrumentation time and cleaning efficacy of manual instrumentation, rotary systems and reciprocating systems in primary teeth: an in vitro study. *J Indian Soc Pedod Prev Dent* 2014; 32(4):311-316.

14. Martinho CM et al. Clinical comparison of the effectiveness of single-file reciprocating systems and rotary systems for removal of endotoxins and cultivable bacteria from primarily infected root canals. *J Endod* 2014; 40(5):625-629.

15. Nekoofar MH et al. Comparison of the effect of root canal preparation by using WaveOne and Protaper on postoperative pain: A randomized clinical trial. *J Endod* Epub ahed of print Feb 23 2015. DOI:10.1016/j.joen.2014.12.026.

16. Gambarini G et al. The influence of three different instrumentation techniques on the incidence of postoperative pain after endodontic treatement. *Ann Stomatol* 2013;4(1):152-155.

17. Koçak S, Koçak MM, Saglam BC, Turker SA, Sagsen B, Er O. Apical extrusion of debris using self-adjusted file, reciprocating single file, and 2 rotary instrumentatio systems. *J Endod* 2013; 39(10):1278-1280.

18. Yared G. A 3-year outcome of endodontic treatments done with the Reciproc® single file canal preparation system. 2012 Disponivel em: <http://www.vdw-dental.com/fileadmin/redaktion/downloads/presse/yared_reciproc_3yearoutcome_en.pdf>

19. Orstavik D, Kerekes K, Eriksen HM. The periapical index: A scoring system for radiographic assessment of apical periodontitis. *Endod Dent Traumatol* 1986; 2: 20-34.

20. Saini HR, Tewari S, Sangwan P, Duhan J, Gupta A. Effect of different apical preparation sizes on outcome of primary endodontic treatement: A randomized controlled trial. *J Endod* 2012;38(10):1309-1315.

21. Pasqualini D, Mollo L, Scotti N, Cantatore G, Castelluci A, Migliaretti G, Berutti E. Postoperative pain after manual and mechanical glide path: A randomized clinical trial. *J Endod* 2012;38(1):32-36.

22. Carvalho FB. Avaliação das alterações radiográficas após tratamento endodôntico de dentes com lesão periapical empregando dois programas de interpretação de imagens. Araraquara-SP;2006. [Dissertação de Mestrado] – Faculdade de odontologia da universidade Estadual Paulista.

# ANEXO I Termo de Consentimento Livre e Esclarecido

Nome do Participante: _________________________________________________

Título da Pesquisa: **Eficácia do sistema Reciproc no tratamento endodôntico de dentes anteriores com lesão periapical: ensaio clínico pragmático randomizado controlado**

Este estudo tem por objetivo avaliar se os resultados dos tratamentos endodônticos realizados com o sistema de lima única Reciporc são iguais aos resultados dos tratamentos realizados com os instrumentos convencionais.

Os sistemas de instrumentação endodôntica com lima única simplificaram os tratamentos endodônticos, tornando-os mais rápidos. Apesar de desejável, é preciso investigar se essa diminuição no tempo do tratamento não lhe compromete os resultados. Especificamente, é preciso saber se o índice de sucesso e a incidência de dor pós-operatória dos tratamentos realizados com esse novo sistema de instrumentação são semelhantes aos dos tratamentos realizados com os sistemas tradicionais.

Os pacientes serão divididos de forma aleatória em dois grupos; em um dos grupos o tratamento endodôntico será realizado com o sistema de limas tradicional enquanto que no segundo grupo o tratamento será realizado com o novo sistema de lima única reciprocante.

Todos os pacientes cujos tratamentos não forem bem sucedidos serão encaminhados e acompanhados para repetição do tratamento – retratamento endodôntico. Aqueles que apresentarem sintomatologia dolorosa após a realização do tratamento serão medicados com nimesulida e devidamente acompanhados e amparados.

Tanto os tratamentos realizados com o sistema tradicional como os realizados com o sistema de lima única reciprocante apresentam risco de não obterem sucesso. Também há o risco de ocorrer sensibilidade dolorosa após o tratamento. Além destes, todos os riscos inerentes à terapia endodôntica tradicional – riscos com a anestesia local separação de instrumentos, ocorrências de sinais e sintomas adversos como inchaço – também existem.

O beneficio dessa pesquisa é de aumentar o conhecimento dos pesquisadores e dos clínicos da área odontologia a cerca desse novo sistema de instrumentação endodôntica, o que pode levar realização de tratamentos endodônticos com maior chance de sucesso e menor tempo de prpcedimento.

Fotografias intra-orais, em que o paciente não é identificado, podem ser realizadas para ilustrar a metodologia e os resultados do estudo. Estas fotografias poderão ser posteriormente apresentadas em publicações e apresentação com finalidades científicas e/ou didáticas.

Os voluntários tem a garantia que receberão, a qualquer momento, respostas a qualquer pergunta e esclarecimento acerca dos procedimentos realizados, aos riscos e benefícios do estudo, bem como outras informações relacionadas a esta pesquisa que julgar importantes. Todas as informações referentes ao tratamento serão estritamente sigilosas.

**Telefone para contato com o pesquisador**

Pesquisador responsável: Universidade Federal de Sergipe

Dra. Maria Amália Ribeiro Gonzaga Ribeiro (79)9 98533200

Pesquisadores:

Dr André Luis Faria e Silva (79) 99142-4251

Fabricio Eneas Diniz de Figueiredo (79) 99127-2996

Universidade Estadual de Montes Claros

Dr Manoel Brito Junior (38) 99952-5069

**Retirada do Consentimento**

O voluntário tem a liberdade de retirar o consentimento a qualquer momento de deixar de participar da pesquisa.

Este documento foi redigido de acordo com as diretrizes e normas que regulamentam as pesquisas envolvendo seres humanos, atendendo as resoluções 466/2012 do Conselho Nacional de Saúde.

Eu, _____________________________________________________________________,

RG:_______________, declaro que, tendo lido todas as informações acima, e suficientemente esclarecido (a) pelo pesquisador ___________________________, estou plenamente de acordo com a realização deste estudo, autorizando, assim, minha participação.

Aracaju, ___ de ____________ de 201__.

____________________________________

Assinatura do pesquisador

____________________________________

Assinatura do Participante
