# Supplementary material for: Apical periodontitis healing and postoperative pain following endodontic treatment with a reciprocating single-file, single-cone approach: A randomized controlled pragmatic clinical trial
Source: PLoS One. 2020 Feb 3;15(2):e0227347. doi: 10.1371/journal.pone.0227347 (PMC6996828; doi:10.1371/journal.pone.0227347)
Supplement: S2 Protocol — (DOCX) [file pone.0227347.s004.docx]

- **Study Protocol**
- **Effectiveness of Reciproc system for anterior teeth with apical periodontitis: a randomized controlled pragmatic trial**
- **Proponent:**
- PhD Student: Fabricio Eneas Diniz de Figueiredo
- Health Sciences Graduate Program
- Federal University of Sergipe
- **Doctoral Adviser:**
- Prof. Dr. André Luis Faria e Silva
- Department of Dentistry
- Health Campus / Biology and Health Science Center
- Federal University of Sergipe
- Aracaju
- June 2015
- **ABSTRACT**
- Single file canal preparation techniques emerged in an attempt to overcome drawbacks related to manual and continuous rotary techniques. It has shown promising results in in-vitro studies, but its effectiveness has not yet been tested in clinical studies. Hence, this proposal seeks to investigate, through an equivalency randomized controlled clinical trial, the effectiveness of the Reciproc® single file, single cone system, regarding its success rate, incidence rate of post-operative pain and PAI score changes. This trial will follow the Consort guidelines, having a parallel design and using an active treatment as control – the crown-down instrumentation technique and the lateral condensation obturation technique. Patients in need of endodontic treatment for anterior teeth due to pulp necrosis and with radiographic evidence of apical periodontitis will be randomized to receive endodontic treatment either by the Reciproc® single file, single cone system or by the crown-down manual technique with stainless steel files. Patients will be questioned about their pain levels at baseline and then 24 hours, 72 hours days and 7 days after treatment is performed. The Wilcoxon test will be used to compare treatment pain scores. Patients will be called for one- year follow-up visits in which PAI score changes and clinical signs of periapical pathosis will be ascertained. Data related to PAI scores will be submitted to the Wilcoxon test to compare treatment outcomes and to the Man-Whitney test to compare one-year follow-up scores within each treatment group. Each individual treatment will be classified as “success” if their PAI scores are 2 or lower and they show no clinical signs or symptoms of periapical disease. Success rates of each treatment and the relative risk of experimental group in relation to control will be determined. In addition, Fishers exact test will be used to compare treatment groups’ success rates. For all analyses, a 95% significance level will be used.
- **Key Words:** Periapical Periodontitis; Root Canal therapy; Randomized Controlled Clinical Trial
- **RESUMO**
- A técnica de instrumentação de canais radiculares com único instrumento reciprocante surgiu como uma tentativa de superar as limitações apresentadas pelas técnicas manuais e de rotação continua. Apesar da facilidade técnica, poucos estudos avaliaram a efetividade do uso de instrumentação reciprocante na prática clínica. Assim, o objetivo desta proposta será avaliar o desempenho clínico da instrumentação reciprocante e obturação com cone único do sistema Reciproc®, no que concerne ao índice de sucesso do tratamento endodôntico e à incidência de dor pós-operatória, através de um ensaio clínico randomizado controlado. Este será realizado seguindo as recomendações do *Consort*, usando controle ativo – instrumentação manual pela técnica coroa-ápice e obturação pela técnica da condensação lateral. Pacientes que apresentem dentes anteriores com quadro clínico de necrose pulpar e evidência radiográfica de periodontite apical serão randomizados para tratamento endodôntico com limas do sistema Reciproc® ou para tratamento com limas manuais. O paciente será questionado qual seu nível de dor percebida antes de iniciar o tratamento e 24 horas, 72 horas e 7 dias após sua realização; os escores serão submetidos ao teste de Wilcoxon para comparação das técnicas endodônticas. Doze meses após o tratamento, os pacientes serão convocados para nova avaliação clinica e radiográfica, na qual serão verificadas alterações no índice PAI com relação ao registrado no *baseline* e sinais ou sintomas clínicos de periodontite periapical. Os dados relativos ao índice PAI serão submetidos ao teste de Wilcoxon para comparação entre as técnicas e ao teste de Man-Whitney para comparar os scores observados na avaliação de 12 meses. O sucesso de cada tratamento será caracterizado se o dente apresentar índice PAI igual ou inferior a 2 e na ausência de sintomatologia clínica. As percentagens de sucesso de cada tratamento e o risco relativo do tratamento experimental em relação ao controle serão calculados. Além disso, as percentagens de sucesso de cada tratamento serão comparadas pelo teste exato de Fisher. Para todas as análises, será utilizado nível de significância estatística de 95%.
- **Palavras-chave:** Periodontite Periapical; Preparo de Canal Radicular; Ensaio Clínico
- **1. Introduction**
- 1.1 Research problem
- One of the main objectives of endodontic therapy is to treat the inflammation of the periapical tissues that is caused by the presence of microbes in the root canal system (RCS) (1). To achieve this objective, the RCS must be disinfected, which is only possible through the cleaning and shaping of the canal with endodontic files and irrigant solutions with antimicrobial properties (2). Traditionally, cleaning and shaping procedures are performed with stainless steel hand files. However, these files have low flexibility, which can result in alteration of root canal anatomy - especially in curved canals (3) - which is not desirable.
- To overcome this problem, nickel titanium files, which present the property of superelasticity, began to be used in rotary motion for root canal preparation. This manner of preparing canals reduces treatment time and the clinicians’ stress, which makes root canal preparation safer and more efficient. However, the main disadvantages of the rotary technique with NiTi files are the high possibility of file separation in the root canal and the necessity of multiple sequences of files to shape the RCS (4,5).
- In an attempt to simplify the root canal preparation phase and reduce the incidence of file breakage, researchers began to the study the preparation efficacy of a single NiTi file in reciprocating motion. The result was the development of the “single-file instrumentation technique”, in which the rotary motion was replaced by the reciprocating motion, mainly to reduce the risk of file breakage (6).
- However, even though it has the advantage of simplicity, the clinical effectiveness and efficacy of the reciprocating single file technique has been poorly studied. Furthermore, results from laboratory studies have raised concern about the technique. First, because it is a very fast technique to execute, reciprocating root canal preparation may hamper the antimicrobial efficacy of the irrigation solution, which is time dependent. Second, recent studies have demonstrated that debris extrusion through the apex foramen related to the reciprocating single file technique is higher than that observed in other techniques (7,8), which may result in higher incidence and severity of post-operative pain.

- 1.2 State of the art
- Dr. Ghassan Yared developed the first reciprocating single file system – the Reciproc system. According to him, the main advantage of the system has is that a single file is required to shape the canal, which makes the technique easy and simple to execute and reduces the risk of cross-contamination (9,10). Furthermore, he also claims that the system presents a lower risk of file breakage. Indeed, many laboratory studies have shown that reciprocating motion enhances the cyclic fatigue resistance of NiTi files, and that reciprocating systems reduce treatment time (11,12,13). However, other laboratory studies have reported that the antibacterial efficacy of reciprocating systems is not superior to that of other systems (14) and that their use may result in a higher extrusion of debris through the apical foramen (7), which can increase the incidence and severity of postoperative pain. This has already been observed in some clinical studies (15, 16). It is important to point out that extrusion of debris through the apex is still a controversial matter (17).
- To the best of our knowledge, there are no controlled clinical studies evaluating the long-term outcomes of endodontic treatment performed with reciprocating single file systems. We found one single study that reported a 93% treatment success rate after reciprocating single file root canal preparation of teeth with apical periodontitis after a period of follow-up of three years. However, this study lacked a control group and was conducted and written by the developer of the system. (18).
- 1.3 Proposal Identification
- This proposal seeks, through a randomized controlled trial, to evaluate the clinical performance of reciprocating single file root canal preparation concerning postoperative pain and the healing of apical lesions.
- **2. Justification**
- A decrease in treatment time may enable a reduction in the cost of treatment, while increasing the productivity of public and private clinics. In addition, it also enhances ergonomics and makes the experience of root canal treatment less uncomfortable for patients. Despite these advantages, faster root canal preparation techniques must not compromise the short and long-term outcomes of root canal treatment. The reciprocating single file philosophy was developed to reduce and simplify root canal treatment time. If its clinical performance is similar or better than the performance of other root canal preparation techniques, it will enhance services delivered to the public, reducing costs and enhancing productivity. Hence, it is important to investigate the clinical performance of endodontic techniques that make use of reciprocating single file root canal preparation in order to help private and public endodontic treatment providers to chose and manage their practice.
- **3. Research Objectives**
- 3.1- General objective
- To evaluate the clinical effectiveness of the Reciproc reciprocating single file system in regards to the treatment of anterior teeth with apical periodontitis
- 3.2. – Specific objectives

1. Compare mean periapical index score (PAI) score changes between reciprocating single file and hand file instrumentation of root canals;
2. Compare the success rate of treatments performed with reciprocating single file and hand file instrumentation of root canals;
3. Compare the incidence and severity of postoperative pain between the two instrumentation modalities;
4. Compare the incidence of flair-up between the two instrumentation modalities

- **4. Goals**

1. Contribute to enhance the scientific knowledge related to the preparation and obturation of root canals with reciprocating single files and the single cone technique, respectively.
2. Contribute to enhance the quality of care offered by public clinics in which endodontic treatment is performed
3. Improve the quality of dentistry-related clinical trials of the post graduate program in Health Sciences of the Federal University of Sergipe, Brazil, considering that these are at the top of the scientific evidence pyramid and, hence, promote evidence based dentistry.
4. Establish partnership with other institutions for the design and execution of multicenter randomized clinical trials;
5. Enhance the quality of education provided to students of the graduate program in Health Sciences of the Federal University of Sergipe;
6. Produce a doctoral thesis and at least two original research articles to be published in journals with an impact factor of 2 or more.
7. **Methodology**

- 5.1 Experimental Design
- This study will be a randomized controlled pragmatic clinical trial, which will follow the CONSORT (<http://www.consort-statement.org/>) statement guidelines. It will have a two-arm, parallel design (1:1 allocation ratio equivalency). The endodontic treatments to be performed are either a combination of a single-file and single-cone (SFSC) technique using the Reciproc system (experimental group) or an association of the crown-down hand files with the lateral compaction obturation (HFLC) technique (control group). The main outcome of interest is mean periapical index score (PAI) (19) difference between the two groups 12 months after treatment. Peak of pain during the first 24 hours after treatment and postoperative pain 24, 48 hours and 7 days after treatment are the secondary outcomes.
- The trial will be based on the following PICO question:
- P (Population): patients above 18 years of age, who are scheduled for an endodontic treatment in one of the public services where the study was performed cities (i.e., Capela, Estância, Laranjeiras and Nossa Senhora do Socorro) located in the state of Sergipe (Brazil), with anterior teeth presenting pulp necrosis and radiographic evidence of apical periodontitis (symptomatic apical periodontitis, asymptomatic apical periodontitis, and chronic apical abscess) with a diameter greater than 2 mm.
- I (Intervention): A combination of a reciprocating single-file and single-cone technique using the Reciproc system.
- C (Control): An association of the crown-down technique hand files with the lateral compaction obturation technique.
- O (Outcomes): Mean PAI difference between the intervention and the control group 12 months after treatment.
- Therefore, the research question is the following: For patients presenting anterior teeth with pulp necrosis and apical periodontitis, is there difference in the outcomes obtained with hand file instrumentation and lateral compaction obturation compared with the outcomes obtained with reciprocating single file instrumentation and single cone obturation, concerning the healing of periapical lesions and postoperative pain incidence and severity? The hypothesis to be tested is that using reciprocating single files instrumentation and single cone obturation has similar effectiveness treating apical periodontitis as hand file instrumentation and lateral compaction obturation technique. In addition, the hypothesis that both endodontic protocols result in the same incidence and severity of postoperative pain and flair-up will also be tested.
- 5.2 Method
- 5.2.1 Sample Size Calculation
- The sample size was calculated for the main outcome, defined as the mean periapical index score (PAI) difference between protocols of the apical lesion 1 year after treatment. The calculation for similarity trial used an equivalence limit of 0.5, a standard deviation of 0.74 (20), type I error of 0.05, and a power test of 0.90. Furthermore, the sample was increased by 20% to compensate for any drop-out. This resulted in 60 teeth per experimental condition.
- 5.2.2 Recruitment and eligibility criteria
- The recruited participants will be patients who are scheduled for an endodontic treatment in one of the public services where the study will take place. Patients with anterior teeth presenting pulp necrosis and radiographic evidence of apical periodontitis (symptomatic apical periodontitis, asymptomatic apical periodontitis, and chronic apical abscess) with a diameter greater than 2 mm will be invited to participate in the study. Those who agreed to participate and sign the informed consent (appendix I) form will be included in this study. Only one tooth per participant will be included in the trial. Teeth with an immature apex, any radiographic evidence of root resorption, previously treated root canal or requiring extensive prosthetic rehabilitation will be excluded. Participants presenting a pre-existing health or oral condition that placed them at risk during the trial, as well as those having generalized periodontal disease, and women who are pregnant or breastfeeding are also going to be excluded.
- 5.2.3 Baseline evaluation
- To evaluate participants’, the pupal condition will be evaluated through thermal sensitivity tests – cold and heat – and through periapical radiographs made by the paralleling technique. A clinical evaluation will also be performed (visual inspection, palpation, vertical and horizontal percussion) to identify and register the presence or absence of sinus tract, tenderness, tooth mobility and pain.
- If the occurrence of pulpal necrosis and apical periodontitis is confirmed and if the patient gives written consent to participate in the study, the baseline PAI will be recorded according with the instructions of Orstavick et al (19).
- Table 1. Periapical Index Score (19).

| - PAI SCORE | - Criteria |
| --- | --- |
| - 1 | - Normal Periapical structures |
| - 2 | - Small changes in bone structure |
| - 3 | - Changes in bone structure with some mineral loss |
| - 4 | - Periodontitis with well-defined radiolucent area |
| - 5 | - Severe periodontitis |

- In case the patient reports any sensitivity before treatment, its level will be registered according to the verbal scale detailed in table 2. If the patient does not report any pain, the score 0 will be registered, which corresponds to absence of pain.
- Table 2. Verbal pain scale (21).

| - 5-Level pain scale to evaluate pain severity: Reference Values given to patients | | |
| --- | --- | --- |
| - 0 | - No pain | - The patient feels good |
| - 1 | - Mild pain | - If distracted, he/she does not fell pain |
| - 2 | - Moderate Pain | - The patient feels moderate pain, even when concentrating on some activity |
| - 3 | - Considerable | - The patients feel very unwell but nevertheless can continue with ordinary activities of daily life |
| - 4 | - Severe pain | - The patient is forced to give up ordinary activities of daily life |
| - 5 | - Extreme | - The patient is no longer able to perform any type of activity and needs to lie down and rest |

- 5.2.4 Random sequence generation and allocation concealment
- A random list will be created using the website [www.sealedenvelope.com](http://www.sealedenvelope.com/). The treatment to be performed on each patient will be placed into opaque and sealed envelopes by a third party not involved in the study intervention. The dentists who will perform the clinical procedures will only open the envelope at the moment of the intervention.
- 5.2.5 Study interventions
- The participants allocated to the experimental intervention group will have their treatment performed by root canal preparation with reciprocating single file (Reciproc system) and obturated with the single cone obturation technique (SFSC group). Participants allocated to experimental control group will have their teeth treated by the crown-down instrumentation technique and the lateral compaction obturation technique (HFLC group).
- 5.2.6 Treatment protocol
- The endodontic treatments will be performed by three endodontists with more than 5 years of clinical experience. After the administration of local anesthesia and the placement of a rubber dam, the carious lesion will be removed and the access cavity performed. All endodontic treatments will be performed in a single session according to the randomization procedure.
- HFLC technique: The glide path will be established with stainless steel hand K-files up to a size #15. Then, the crown-down technique will be performed, initially with Gates-Glidden burs, which are to be used in a step-down manner to enlarge the orifice, prepare the cervical and middle-thirds of the canal, and provide straight-line access to its apical third. The apical foramen will be located by using an electronic apex locator (RomiApex A-15 Romidan, Kiryat Ono, Israel), and the working length established 1.0 mm short of its “0.0” reading. Apical preparation will be performed using ISO stainless steel hand files (Dentsply Sirona Endodontics, Ballaigues, Switzerland), starting with the selection of the first file to bind at the working length. The final instrumentation file will be set at 3 sizes larger than the first file used. The lateral compaction obturation technique will be used to fill the canals. A .02 taper gutta-percha cone will be selected according to the master apical file and then prefitted into the canal at the working length. After the canal is dried with paper points (Dentsply Sirona Endodontics), the master cone will be lightly coated with an epoxy resin-based sealer (AH Plus, Dentsply DeTrey, Konstanz, Germany) and placed into the canal down to the working length. Lateral compaction will be performed with finger spreaders (Dentsply Sirona Endodontics) and accessory cones (Dentsply Sirona Endodontics) chosen according to the final root canal dimensions. The excess filling material will be removed with a heated instrument and the access cavity sealed with glass-ionomer.
- SFSC technique: Root canal preparation will be performed with Reciproc instruments (VDW GmbH, Munich, Germany), following the instructions of Yared (8). The Reciproc file selection will be based on a preoperative radiograph and root canal space. If the canal is partially or completely invisible on the radiograph, an R25 file will be selected. Otherwise, a #30 or #20 hand file will be inserted passively to 2/3 of the estimated working length. An R50 file will be selected whenever a #30 hand file reached this length, and an R40 file whenever the 2/3 are reached by a #20 hand file. R25 will be selected for narrow canals. The Reciproc instrument will be introduced into the root canal with a slow in-and-out pecking motion, which does not exceed 3-4 mm in amplitude. After three in-and-out movements, the file will be pulled out of the canals to clean the flutes. When the instrument reaches 2/3 of the estimated working length, the foramen will be located by using an electronic apex locator (RomiApex A-15), and the actual working length was established 1.0 mm short of the “0.0” electronic reading. Finally, the Reciproc instrument will then be reused in the same manner until the working length is reached. After canal preparation, a matching-taper single gutta-percha cone (VDW GmbH, Munich, Germany) will be selected according to the file used to instrument the canal. The canal will be dried with sterile paper points (Dentsply Sirona Endodontics), and the selected cone was lightly coated with AH plus sealer and placed into the canal down to the working length. The excess filling material will then be removed with a heated instrument, and the access cavities sealed with glass-ionomer.
- All teeth from both groups will be submitted to the following procedures: irrigation with 2.5% sodium hypochlorite; canal patency by passing a stainless steel K-file ≤ #15 approximately 1.0 mm beyond the working length; smear layer removal with 17% EDTA for 3 minutes; and final restoration with composite resin. Pre- and post-operative radiographs will be taken with Kodak UltraSpeed #2, D sensitivity film (Kodak, São Paulo, SP, Brazil), processed manually by the time/temperature method. The long cone parallel technique will be used by employing X-ray holders (Endo Rh plus, Indusbello, Londrina, PR, Brazil), which are to be placed on the 30.5 x 40.5 mm size film, parallel to the long axis of the tooth and perpendicular to the X-Ray.
- 5.2.8 Evaluations
- Thirty periapical radiographs of endodontically treated teeth not included in the study will be used for calibration procedures by two independent and blinded evaluators. The evaluation of such radiographs will be repeated until intra- and inter-evaluator agreement has a Kappa coefficient higher than 0.80.
- Apical periodontitis will be classified using the initial radiography, and according to PAI scores. Bitewing film holders will be used to standardize the position of radiographs. Silicone impression material (Optosil Comfort, Heraeus Kulzer, Hanau, Germany) will be placed on the film holder, and impressions of the tooth undergoing treatment will be taken. Then, the impression will be used to place the device in the same position during the follow-up evaluations.
- The patients will be called after 12-months. New periapical radiographs will be taken, and the lesions will be reclassified according to the PAI scale. Then, the teeth will be classified as healed (scores 1 or 2); healing (reduced score compared to baseline, but higher than 2); or not healed. Teeth that are clinically asymptomatic – defined by absence of pain, tenderness to percussion and/or palpation, sinus tract, or soft tissue swelling – and present a PAI score of 1 or 2 will be classified as “success”.
- If the patients record any postoperative pain, it will be recorded using both a visual analog scale (VAS) and a verbal rating scale (VRS). For the VAS, the patient will set her/his pain level by pointing (with a pen) along a 10-cm continuous line between two endpoints (ranging from the absence of pain to unbearable pain). The distance between the marking and the border corresponding to the absence of pain will be recorded. The peak of pain at the first 24 h, as well the pain reported at 24 h, 72 h and 7 days after the end of the endodontic treatment will be recorded. Postoperative pain was also scored according to the VRS. The latter scale was used to assess the incidence of postoperative pain (scores differing from 0). The occurrence of flare-up was recorded when the patients presented severe pain and swelling following the endodontic treatment.
- 5.2.8 Blinding
- Because this is an intervention study, clinicians cannot be blinded for the procedures they will perform. The patients will not be informed of which group they are allocated to.
- 5.3 Statistical analysis
- Baseline demographic data of the participants and clinical characteristics of the teeth included in the study will be categorized, and the absolute and relative frequencies calculated (except for PAI scores, final preparation size scores, age and follow-up time). The difference between the interventions regarding the distribution of these characteristics will be assessed by Chi-squared analysis. For PAI scores, final preparation size scores, age and follow-up time, data will be analyzed using either t-test or Mann-Whitney Rank-Sum test.
- The Wilcoxon Rank-Sum test for equivalence using two one-sided tests (TOST) procedure will be used to assess if, 12 months after the treatment, the 95% confidence intervals of the mean PAI score treatment difference falls within the pre-established equivalence limit (main outcome of interest). For all other outcomes, traditional two-sided superiority analyses will be used. The association between treatment protocol and changes on periapical status will be analyzed by Chi-squared test or by Fisher`s exact test. The success rates for each treatment protocol will be calculated, and differences analyzed by Chi-squared testing. Univariate logistic regression will used to determine any association between explanatory variables and the treatment’s success rate. Factors with a p-value < 0.1 will be included in a multivariate analysis. Odds ratios and 95% confidence intervals will be calculated.
- For all VAS data, normal distribution and possible differences between the treatments will be assessed through the Shapiro-Wilk test and t-test, respectively. Data from VRS will be analyzed through the Wilcoxon rank-sum test. The incidence of postoperative pain and flare-up will be calculated, and Chi-squared tests will be used to assess any possible association between treatment and incidence. Differences between the treatments regarding the incidence and intensity of postoperative pain will be calculated, as well as their confidence intervals at 95%. The level of significance will be set at 95% for all analyses.
- .
- **6. Scientific contributions of the proposal**
- This proposal will enhance the knowledge root canal preparation systems, which will help to improve education and practice of Clinical Endodontics. In addition, it can help the management process of public and private clinics that provide Endodontic care. Considering that randomized controlled trials provide the highest level of scientific evidence to support clinical practice – because of their capacity to control confounding variables – we expect that the results of this trial will contribute to evidence based practice of Endodontics and Dentistry.
- **References**
- 1.Trope M. The vital tooth – its importance in the study and practice of endodontics. *Endo Topics* 2003;5:1 .
- 2. Siqueira Jr JF, Roças IN. Microbiology and treatment of endodontic infections. In: Hargreaves KM, Cohen S. Pathways of the pulp. 10 Ed. St Louis: Mosby Elsevier, 2001;559-600
- 3. Pereira HSC, Silva EJNL, Coutinho-Filho TS. Movimento Reciprocante em Endodontia: Revisão de Literatura. *Rev bras odontol* 2012;69(2):246-249.
- 4. Yared G. Canal preparation with nickel-titanium or stainless steel instruments without the risk of instrument fracture: prelimirary observations. *Restor Dent Endod* 2014.
- 5. Peters OA. Current challenges and concepts in the preparation of root canal systems: A review. *J Endod* 2004; 30(8):559-567.
- 6.Çapar I, Arslan H. A review of instrumentation kinematics of engine-driven nickel-titanium instruments.’ Accepeted article’, doi:10111/iej.12432
- 7. Burklein S, Schafer E. Apically extruded debris with reciprocating single-file and full-sequence rotary instrumentation systems. *J Endod* 2012; 38(6): 850-852.
- 8. Nayak G, Singh I, Shetty S, Dahiya S. Evaluation of apical extrusion of debris and irrigant using two new reciprocating and one continues rotation single single file system. *J Dent (Tehran)* 2014; 11(3):302-309.
- 9. Yared G. Canal preparation with only one Ni-Ti rotary instrument: preliminar observations. *Int Endod J* 2008; 41:339-344.
- 10. Yared G. Canal Preparation with only one Reciprocating Instrument without prior hand filing. A new concept. 2001. Disponível em: <http://www.vdwreciproc.de/images/stories/pdf/GY_Artikel_en_WEB.pdf>.
- 11. Frota MF et al. Comparison of cyclic fatigue and torsional resistance in reciprocating single-file systems and continuous rotary instrumentation systems. *J Oral Sc* 2014; 56(4):269-275.
- 12.Kiefner P, Ban M, De-Deus G. Is the reciprocating movement per se able to improve the cyclic fatigue resistance of instruments? *Int Endod J* 2014; 47(5):430-436.
- 13. Katge F, Patil D, Poojari M, Pimpale J, Shitoot A, Rusawat B. Comparison of instrumentation time and cleaning efficacy of manual instrumentation, rotary systems and reciprocating systems in primary teeth: an in vitro study. *J Indian Soc Pedod Prev Dent* 2014; 32(4):311-316.
- 14. Martinho CM et al. Clinical comparison of the effectiveness of single-file reciprocating systems and rotary systems for removal of endotoxins and cultivable bacteria from primarily infected root canals. *J Endod* 2014; 40(5):625-629.
- 15. Nekoofar MH et al. Comparison of the effect of root canal preparation by using WaveOne and Protaper on postoperative pain: A randomized clinical trial. *J Endod* Epub ahed of print Feb 23 2015. DOI:10.1016/j.joen.2014.12.026.
- 16. Gambarini G et al. The influence of three different instrumentation techniques on the incidence of postoperative pain after endodontic treatement. *Ann Stomatol* 2013;4(1):152-155.
- 17. Koçak S, Koçak MM, Saglam BC, Turker SA, Sagsen B, Er O. Apical extrusion of debris using self-adjusted file, reciprocating single file, and 2 rotary instrumentatio systems. *J Endod* 2013; 39(10):1278-1280.
- 18. Yared G. A 3-year outcome of endodontic treatments done with the Reciproc® single file canal preparation system. 2012 Disponivel em: <http://www.vdw-dental.com/fileadmin/redaktion/downloads/presse/yared_reciproc_3yearoutcome_en.pdf>
- 19. Orstavik D, Kerekes K, Eriksen HM. The periapical index: A scoring system for radiographic assessment of apical periodontitis. *Endod Dent Traumatol* 1986; 2: 20-34.
- 20. Saini HR, Tewari S, Sangwan P, Duhan J, Gupta A. Effect of different apical preparation sizes on outcome of primary endodontic treatement: A randomized controlled trial. *J Endod* 2012;38(10):1309-1315.
- 21. Pasqualini D, Mollo L, Scotti N, Cantatore G, Castelluci A, Migliaretti G, Berutti E. Postoperative pain after manual and mechanical glide path: A randomized clinical trial. *J Endod* 2012;38(1):32-36.
- 22. Carvalho FB. Avaliação das alterações radiográficas após tratamento endodôntico de dentes com lesão periapical empregando dois programas de interpretação de imagens. Araraquara-SP;2006. [Dissertação de Mestrado] – Faculdade de odontologia da universidade Estadual Paulista.
- Appendix I
   Consent Form
- Participants’ Name _________________________________________________
- Research Title: **Effectiveness of Reciproc system for anterior teeth with apical periodontitis: a randomized controlled pragmatic trial**
- This study seeks to evaluate if endodontic treatment outcomes obtained with reciprocating single files are similar to those obtained with conventional files.
- Reciprocating single file systems have simplified endodontic treatment, making it faster to execute. Even though this is desirable, the effect of this time reduction on the outcomes of the treatment must be investigated. Specifically, it is important to determine if postoperative pain and the healing of apical periodontitis resulting from treatments performed with reciprocating single files are similar to those obtained with traditional file systems.
- The participants of this study will be randomly assigned to one of two groups: 1) endodontic treatment performed with traditional hand files, 2) endodontic treatment performed with reciprocating hand files.
- All participants whose treatments are not successful will have their treatments redone (retreatment procedure). Those who present postoperative pain will be treated with analgesic drugs (Ibuprofen) and followed-up until they are free of symptoms.
- Both treatment protocols present risk of failure. In addition, the occurrence of postoperative pain at some level is expected. Besides these, all undesirable events related to endodontic treatment (local anesthesia risks, file separation, flair-up) also have the risk of occurring.
- The main benefit of this research is the knowledge to be gained by researchers and clinicians regarding these new root canal preparation files, which will help the development of new endodontic protocols that are less time-consuming and result in similar or better outcomes.
- Intra-oral photographs in which the participant is not identified may be taken to illustrate the methods and/or the results of the study. These photographs may also be presented in publications and presentations with scientific or educational ends.
- The participants have the guarantee they will receive, at any moment, any answers and clarification they require about procedures, risks and benefits of this study as well as other information related to this research they find important. All information related to the treatment and the participants are strictly confidential
- **Researchers’ contact**
- Main Researcher: Federal University of Sergipe
- Dr André Luis Faria e Silva (79) 99142-4251
- Researchers:
- Dra. Maria Amália Ribeiro Gonzaga Ribeiro (79) 9 98533200
- Fabricio Eneas Diniz de Figueiredo (79) 99127-2996
- Montes Claros State University
- Dr Manoel Brito Junior (38) 99952-5069
- **Withdrawal of Consent**
- The participant has the right to withdrawal consent and leave the research at any moment he/she desires
- This document was written in accordance with the guidelines and norms that regulate research with human subjects, respecting resolution 466/2012 of the National Council of Health.
- I, _____________________________________________________________________,
- ID:_______________, declare I have read all above information and am sufficiently clarified by researcher _____________________________________ ___________________________, and that I agree with the conduction of this study , thus, authorizing my participation in it.
- Aracaju, ___ de ____________ de 201__.
- ____________________________________
- Researcher’s Signature
- ____________________________________
- Participant’s signature
